# Comparison of fMRI paradigms assessing visuospatial processing: Robustness and reproducibility

**DOI:** 10.1371/journal.pone.0186344

**Published:** 2017-10-23

**Authors:** Verena Schuster, Peer Herholz, Kristin M. Zimmermann, Stefan Westermann, Stefan Frässle, Andreas Jansen

**Affiliations:** 1 Laboratory for Multimodal Neuroimaging, Department of Psychiatry, University of Marburg, Marburg, Germany; 2 Department of Clinical Psychology and Psychotherapy, University of Bern, Bern, Switzerland; 3 Translational Neuromodeling Unit (TNU), Institute for Biomedical Engineering, University of Zurich & ETH Zurich, Zurich, Switzerland; 4 Core-Unit Brainimaging, Faculty of Medicine, University of Marburg, Marburg, Germany; Centre de neuroscience cognitive, FRANCE

## Abstract

The development of brain imaging techniques, in particular functional magnetic resonance imaging (fMRI), made it possible to non-invasively study the hemispheric lateralization of cognitive brain functions in large cohorts. Comprehensive models of hemispheric lateralization are, however, still missing and should not only account for the hemispheric specialization of individual brain functions, but also for the interactions among different lateralized cognitive processes (e.g., language and visuospatial processing). This calls for robust and reliable paradigms to study hemispheric lateralization for various cognitive functions. While numerous reliable imaging paradigms have been developed for language, which represents the most prominent left-lateralized brain function, the reliability of imaging paradigms investigating typically right-lateralized brain functions, such as visuospatial processing, has received comparatively less attention. In the present study, we aimed to establish an fMRI paradigm that robustly and reliably identifies right-hemispheric activation evoked by visuospatial processing in individual subjects. In a first study, we therefore compared three frequently used paradigms for assessing visuospatial processing and evaluated their utility to *robustly* detect right-lateralized brain activity on a single-subject level. In a second study, we then assessed the *test-retest reliability* of the so-called Landmark task–the paradigm that yielded the most robust results in study 1. At the single-voxel level, we found poor reliability of the brain activation underlying visuospatial attention. This suggests that poor signal-to-noise ratios can become a limiting factor for test-retest reliability. This represents a common detriment of fMRI paradigms investigating visuospatial attention in general and therefore highlights the need for careful considerations of both the possibilities and limitations of the respective fMRI paradigm–in particular, when being interested in effects at the single-voxel level. Notably, however, when focusing on the reliability of measures of hemispheric lateralization (which was the main goal of study 2), we show that hemispheric dominance (quantified by the lateralization index, LI, with |LI| >0.4) of the evoked activation could be robustly determined in more than 62% and, if considering only two categories (i.e., left, right), in more than 93% of our subjects. Furthermore, the reliability of the lateralization strength (LI) was “fair” to “good”. In conclusion, our results suggest that the degree of right-hemispheric dominance during visuospatial processing can be reliably determined using the Landmark task, both at the group and single-subject level, while at the same time stressing the need for future refinements of experimental paradigms and more sophisticated fMRI data acquisition techniques.

## 1. Introduction

Hemispheric specialization is a fundamental principle of human brain organization and describes the fact that different cognitive or executive processes are distributed differently across the two hemispheres of the brain. Such functional asymmetries between the hemispheres have been known since the mid-19^th^ century [1–3], and their study has been made more widely feasible with the development of modern brain imaging techniques. In particular, functional transcranial Doppler sonography (fTCD) and functional magnetic resonance imaging (fMRI) for the first time enabled non-invasive studies of the hemispheric lateralization of cognitive functions in large cohorts of both patients and healthy subjects [4]. Using these methods, researchers have mapped the hemispheric lateralization of various cognitive functions and highlighted that for all these processes, the degree of lateralization is subject to inter-individual variability. For instance, while most people show left-hemispheric dominance for language, an atypical right-hemispheric or bilateral form of language lateralization has been observed in up to 10% of the human population [5–9]. On the contrary, visuospatial attention is lateralized predominantly to the right hemisphere, again subject to marked variability across subjects [10–12].

A thorough analysis of hemispheric lateralization necessitates systematic analyses of these inter-individual differences. For this, experimental paradigms must provide robust measures of hemispheric lateralization not only at the group level, but also in individual subjects [13–15]. This refers to the test-theoretical concept of test-retest reliability (or within-subject stability) and has been studied for various lateralized cognitive processes, such as language [14,16], face processing [17], motor processing [18–20] and declarative memory [13]. However, the reliability of imaging paradigms assessing a typical right-lateralized cognitive function, i.e., visuospatial processing, has received considerably less attention so far.

The aim of the present study was therefore to establish a paradigm that *robustly* and *reliably* evokes right-hemispheric dominance of fMRI activation patterns both at the group and single-subject level (note that robustness and reliability are distinct test-theoretical concepts and a definition of both metrics is given in the Methods section). Here, we focused on visuospatial processing as a “typical” right-lateralized cognitive process, in line with an extensive body of previous literature, e.g. [10–12] (but see the Discussion for a critical review of this assumption). Specifically, we compared three paradigms (“dots-in-space” task, mental rotation task, and Landmark task), that had been used frequently in imaging studies to determine hemispheric dominance and evaluated their respective utility to provide *robust* and *reliable* estimates of right-hemispheric lateralization. In what follows, we briefly introduce the different paradigms.

First, we used the “dots-in-space” task, which has been described as a “robust and reliable method for investigating laterality of visuospatial skills” in a previous fTCD study [21]. In the “dots-in-space” task, participants have to memorize the location of circles, which are randomly distributed on a black screen. This primarily assesses spatial memory skills, which was found to engage a right-hemispheric brain network [22], comprising areas in the ventrolateral frontal cortex, occipital cortex, parietal cortex and premotor cortex [23]. However, until now, this paradigm has not been translated into an fMRI setting.

Second, the mental rotation task was implemented, testing a second aspect of visuospatial abilities, namely spatial orientation. The mental rotation task has been used previously both in fTCD (e.g., [24]) and fMRI studies (e.g., [25]), however, yielding contradictory results with regard to the evoked lateralization. For example, Dorst and colleagues found right-lateralized activation in the majority (72.4%) of participants [24]. In contrast, Hattemer and colleagues did not observe significant lateralization to the right hemisphere using either fTCD or fMRI, but bilateral activation in the middle and superior frontal gyrus, the insular cortex, thalamus, mesencephalon and cerebellum [26].

Third, we tested the Landmark task, which is often used in fMRI studies that investigate spatial attention, representing another important aspect of visuospatial abilities. The Landmark task originates from a clinical setting, where it served as a bedside test of hemi-spatial neglect. The underlying cognitive processes involved in the Landmark task are summarized in Ciςek et al. [27] and include spatial judgments, sustained attention and object-based spatial processing, leading to an activation of the dorsal attention network.

Hence, all three fMRI tasks involve somewhat distinct aspects of visuospatial abilities (e.g., spatial memory, spatial orientation, spatial attention), but have all been linked to an increased recruitment of right-hemispheric brain regions. As the aim of the present study was to establish an fMRI paradigm that robustly and reliably evokes right-hemispheric lateralization in the human brain, we compared these different right-hemispheric lateralized cognitive processes under the umbrella term of visuospatial processing.

The present study was divided into two parts. In the first part (“study 1”), we focused on the comparison of the above-mentioned imaging paradigms for studying visuospatial processing: the “dots-in-space” task (adapted from [28]), the mental rotation task [24] and the Landmark task [11]. The aim of study 1 was to test which of these three paradigms was able to robustly map right-hemispheric dominance. In the second part (“study 2”), we then assessed the test-retest reliability of the paradigm that yielded the most robust results in study 1.

## 2. Methods

### 2.1 Subjects

Sixteen subjects (6 men, mean age: 24.7 ± 2.5 years) participated in study 1 (comparison of imaging paradigms). Notably, one subject had to be excluded from study 1 because of uncomfortableness in the scanner, yielding 15 remaining subjects. In study 2 (assessment of test-retest reliability), 20 subjects (10 men, mean age: 25.0 ± 2.2 years) participated. To investigate the test-retest reliability of the imaging paradigms, all subjects in study 2 underwent the identical experiment twice in two separate sessions. The time interval between sessions ranged from 5 to 8 days (mean time interval: 6.9 ± 0.2 days). All 36 subjects were right-handed, had completed the equivalent of a high school degree (“Gymnasium”) and were native German speakers. None had any history of medical, neurological or psychiatric illnesses or brain pathology. All subjects had normal or corrected to normal vision. Each gave informed written consent prior to participation. The study conformed with the Declaration of Helsinki and was approved by the local ethics committee of the Medical Faculty of the University of Marburg.

### 2.2 Experimental paradigms

In study 1, subjects performed three different tasks, which are well established for testing hemispheric lateralization during spatial processing (see below for a detailed description of each task): “dots-in-space” task, adapted from [21], mental rotation task [24], and Landmark task [11]. The order of these tasks was pseudorandomized and counterbalanced across subjects. The aim of study 1 was to identify which of these paradigms evoked robust right-hemispheric lateralization both at the group and single-subject level. In study 2, we then evaluated the test-retest reliability for the most robust paradigm (as quantified by our pre-defined criteria of robustness described below). All paradigms were implemented and displayed using the Presentation® Software package (Version 14.1, http://neurobs.com). Prior to the experiment, subjects practiced each task outside the MR-scanner to ensure that they had understood the instructions. During the fMRI measurements, responses were reported by pushing a button on an MR-compatible response box, which was located on the left and right thigh.

**Fig 1:**
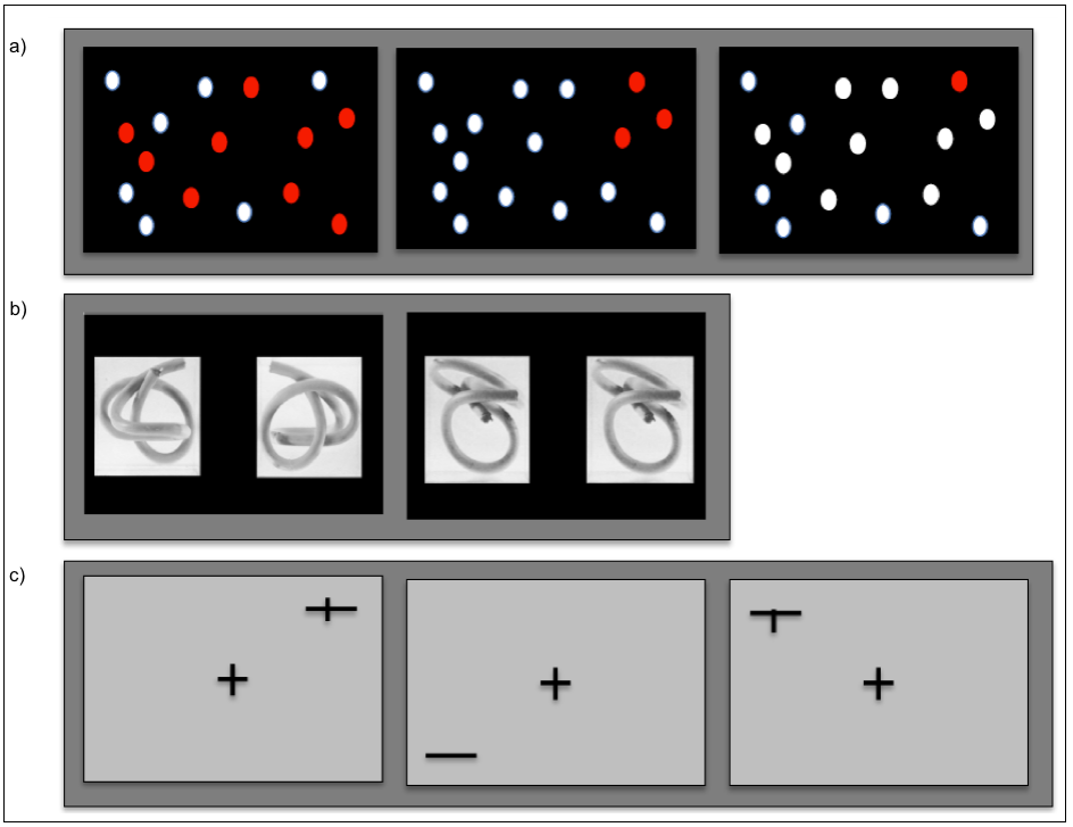
fMRI-Stimuli. a) “Dots-in-space” task: Activation (left, middle) and control-condition (right) of the “dots-in-space” task. In the activation condition subjects were asked whether they have seen the same arrangement of red dots during the encoding part. In the control condition, they were asked to decide whether there was a red dot or not. b) Mental rotation task: Activation (left) and control-condition (right) of the mental rotation task. In the activation condition subjects were asked to indicate, whether the figure on the right, showed the figure on the left from behind, the bottom, the top, the left, or the right by pressing the respective finger of their right hand. In the control condition, they were asked to decide whether the two figures were identical or not. All stimuli were taken from Stumpf and Fay (1983), scanned in and corrected for contrast and brightness differences. c) Landmark task: Activation (left) and control-condition of version A (middle) and version B and C (right) of the Landmark task. During the activation condition, subjects were asked to decide whether the horizontal line was transected left or right from the middle (version A) or whether the line was bisected correctly (version B and C). The horizontal line, which measured 200 pixels (13.48 cm), appeared 0.6 s in one of the four corners of the screen. In versions A and B, the vertical line was centered either exactly in the middle of the horizontal line or slightly deviated to the left or the right. Distances of 15, 30 and 45 pixels (resulting in 1.01, 2.02, and 3.03 cm lengths and visual angles of 0.241°, 0.482° and 0.723° respectively) were used to shift the vertical line to either side. Version C was characterized by smaller distance variations. Distances to the middle of the horizontal line were 12, 25 and 37 pixels, resulting in 0.809, 1,685 and 2.493 cm and visual angles of 0.193°, 0.402° and 0.505° respectively. In the control condition of version A (middle) subjects were asked to decide, whether a transecting line was present, whereas in the control condition of version B and C, subjects had to decide whether the vertical line transected the horizontal one or not (right figure). Answers were indicated with the index- and middle finger of the right hand (version A) or of both hands (version B and C).

“Dots-in-space” task: The “dots-in-space” task used in the present study was based on a spatial memory task originally developed for fTCD [21]. Subjects had to memorize the location of a number of red dots randomly interspersed with a larger number of white dots, presented on a black background (Fig 1). The dots were randomly distributed across the screen and not aligned in rows or columns to prevent verbal encoding strategies. The task was divided into two parts: an encoding phase and a retrieval phase. Subjects were shown 20 different arrangements of white and red dots (“target stimuli”). Each target stimulus was shown three times in a pseudorandomized order. Ten of the target stimuli consisted of 17 white and 9 red dots (“difficult condition”), whereas the remaining target stimuli consisted of 23 white and 3 red dots (“easy condition”). During the encoding phase, each stimulus was presented for 5 s and then followed by a blank screen with an inter-stimulus interval (ISI) of 1 s. Subjects were instructed to memorize the location of the red dots.

**Fig 2:**
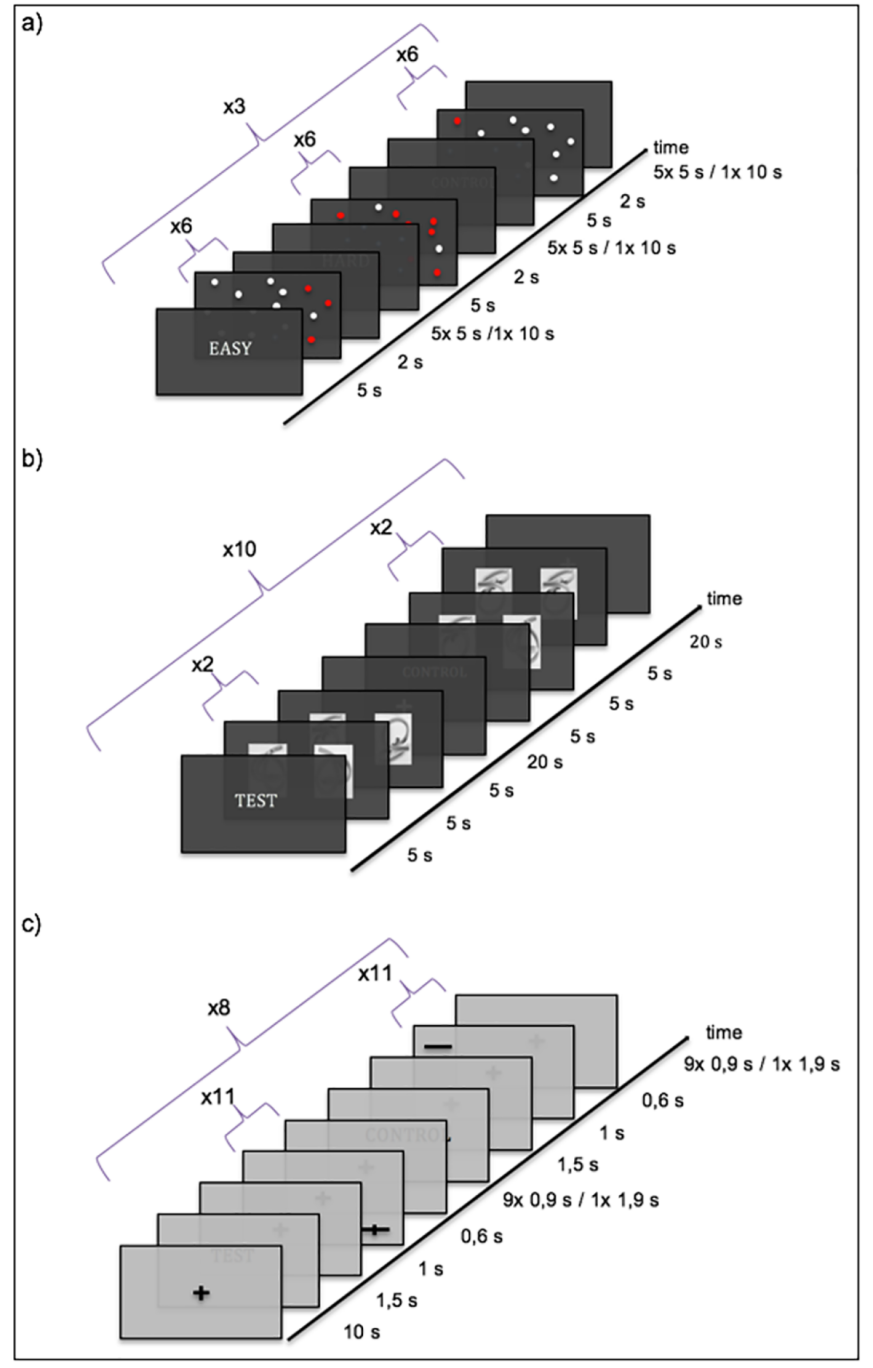
Schematic representation of the experimental procedure. a) “Dots-in-space” task: Each experimental trial began with an introduction screen (5 s) indicating the following condition (easy, hard, control). Participants were asked, whether they have seen the same arrangement of dots during the encoding part or not (easy, hard), or whether the arrangement contained a red dot (control) by pressing a button with the index- or middle finger of their right hand, respectively. The paradigm consisted of three blocks of each condition, with six stimuli within each block. b) Mental rotation task: Each experimental trial began with an introduction screen (5 s) indicating the following condition (test, control). Participants were asked, to decide whether the picture on the right side showed the identical cube on the left side seen from the left, right, back, top or bottom. Answers were indicated by pressing either their right thumb (from the left), index finger (from the bottom), middle finger (from the top), ring finger (from the back) or the little finger (from the right). During the control condition (low spatial load), either pairs of identical cubes shown from the same perspective or two different cubes were presented. Subjects had to decide whether the presented cubes were the same or not. Subjects gave their answer by pressing a button with the index finger (same) or middle finger (different) of their right hand on a MR-compatible response box. The paradigm consisted of 10 control and 10 activation blocks that appeared in pseudorandomized order. In each block 4 stimuli were shown. Each stimulus was presented for 5 s. c) Landmark task: The paradigm began with a fixation screen (10 s) prior the experimental conditions. Participants were instructed to fixate the cross during the whole experiment. Each experimental trial began with an introduction screen (1.5 s) indicating the following condition (test, control). In version A participants were asked, whether the horizontal line, appearing in one of the four corners of the screen, was intersected left or right from the middle point (test) or whether a transection mark was present or not (control). Answers were indicated by pressing a respective button with their right hand. In version B and C participants were asked to decide whether the horizontal line was bisected correctly or not. In the control condition, they were asked to indicate, whether the horizontal line was transected by the vertical one or not. Answers were indicated by pressing the respective button with both hands, simultaneously.

Functional images were acquired only during the retrieval phase, which consisted of 18 blocks (Fig 2A). Six blocks belonged to the difficult condition, six blocks to the easy condition (i.e., test conditions), and six blocks to a control condition (see below). Again, the order of conditions was pseudorandomized. Each block started with a black screen (3 s) followed by an instruction screen that indicated the task condition of the upcoming trial (5 s). After the instructions, six stimuli (“test stimuli”) were presented. Each test stimulus was shown for 2 s followed by a jittered ISI (average length 1 s; range: 0.6 s to 1.4 s) and a black screen after the sixth stimulus (5 s). This resulted in a total block length of 30 s. During the retrieval phase, subjects were asked to decide whether the presented test stimulus was familiar or not. Subjects responded by pressing a button with the index finger (yes) or middle finger (no) of their right hand on the MR-compatible response box. During the control condition, subjects were instructed to decide whether a stimulus contained exactly one red circle or not. Stimuli in this condition consisted of either only white dots or white dots and exactly one red dot randomly located on the black background (Fig 1A). The total length of the paradigm was 9 minutes 10 seconds.

Mental rotation task: The mental rotation task used in the present study was based on a spatial orientation task that had also been originally introduced for fTCD [24,25]. Subjects were presented with pairs of three-dimensional images of transparent cubes with 1, 2 or 3 cables inside showing the same object from two different perspectives (Fig 1B). During the activation condition (high spatial processing load), subjects were presented with pairs of identical cubes, however, the right-sided cube was always seen from different perspectives. Subjects were asked to decide whether the right-sided cube showed the left-sided cube seen from the left, right, back, top or bottom. According to the perspective in which the right cube was presented, answers were indicated by pressing either the right thumb (perspective: left), index finger (bottom), middle finger (top), ring finger (back) or the little finger (right). During the control condition (low spatial processing load), either pairs of identical cubes shown from the same perspective or two different cubes were presented. Subjects had to decide whether the presented cubes were identical or not. Subjects responded by pressing a button with the index finger (same) or middle finger (different) of their right hand. The paradigm consisted of 10 control and 10 activation blocks that were presented in a pseudorandomized order (Fig 2B). In each block, 4 stimuli were shown for 5 s each. Before each block, an instruction screen informed the subjects about the condition of the upcoming block (5 s). Each block was followed by a baseline period of 20 s, resulting in a total block length of 45 s. The total length of the paradigm was 15 minutes.

Landmark task: The Landmark task has been used frequently to study spatial attention by means of functional imaging [11,14,29,30]. The paradigm consisted of two conditions. In the activation condition (high spatial processing load), subjects had to decide whether a horizontal line was correctly bisected by a crossing vertical line or not. In the control condition (low spatial processing load), subjects had to decide whether a horizontal line contained a transection mark (irrespective of the position of that mark) or not (Fig 1C). Eight activation and eight control blocks were presented in an alternating order. Each block lasted 20 s and contained 11 stimuli that were presented for 0.6 s followed by an ISI of 0.9 s. Stimuli were presented in the four corners of the screen while subjects had to fixate the center of the screen. This prevented subjects from solving the task by simply attending a single point as the center of all lines without the need to engage in spatial processing. Each block was preceded by an instruction screen displayed for 1.5 s, informing subjects about the condition of the upcoming block. The total length of the paradigm was 5 minutes 34 seconds.

We used three different versions of the Landmark task (version A in study 1, version B and C in study 2). We slightly adapted the task in study 2 for the following two reasons: First, although the Landmark task was the most robust paradigm in study 1 according to our criteria (see below), the BOLD signal difference between activation and control condition was relatively small, as expressed, e.g., by low t-values. One reason for this might have been that the activation task was too simple, as indicated by the behavioral results. We therefore made the task more difficult in version C by using more demanding visual stimuli in the activation condition (Fig 1C). Second, we adapted both the control stimuli and the instructions (see below) to make our version of the Landmark task more similar to those used in recent work (e.g., [16,31]). Additionally, consistent with previous studies [11,27], proper fixation of the subjects was explicitly controlled in versions B and C by online visual inspection of the recorded traces of the direction of eye gaze using an MRI-compatible infrared-sensitive camera (EyeLink 1000, SR Research, Osgoode, ON, Canada). Specifically, a qualitative screening of the eye-tracking data was performed to identify subjects that poorly fixated the central cross during the experiment (e.g., performing saccades to the presented stimulus) and should thus be excluded. Importantly, no such cases were observed and thus all subjects were included in the subsequent analyses. Note that eye-tracking data in the present study did not enter any further analysis.

In version A, we used horizontal lines with or without a transection mark as control stimuli (Fig 1C left and middle) and subjects had to decide whether the transection mark was present or not (irrespective of the position of that mark). In the activation condition, subjects had to decide whether the horizontal line was transected left or right from the middle or whether the vertical line crossed the horizontal line on the left or right side. For both conditions, subjects reported their decision with either their right index (“right side” or “transection mark is present”) or middle finger (“left side” or “no transection mark”). In versions B and C, all stimuli contained a vertical line. In the control condition, the vertical line crossed the horizontal line in half of the images, whereas the vertical line was above or beneath the horizontal line for the other half (Fig 1C right). Subjects had to decide whether the vertical line crossed the horizontal line or not. In the activation condition, subjects had to decide whether the horizontal line was correctly bisected or not. For both conditions, subjects reported their decision using both hands. Specifically, they indicated their answer by pressing both index (“correctly bisected” or “vertical line transects”) or middle fingers (“not correctly bisected” or “vertical line does not transect”) simultaneously.

### 2.3 MRI data acquisition

Subjects were scanned on a 3-Tesla TIM-Trio MR Scanner (Siemens Medical Systems) with a 12-channel head matrix receive coil at the Department of Psychiatry and Psychotherapy, University of Marburg. Functional images were acquired using a T_2_*-weighted echo planar imaging (EPI) sequence sensitive to the Blood Oxygen Level Dependent (BOLD) contrast. Slices covered the whole brain and were positioned transaxially parallel to the anterior-posterior commissural line (AC-PC). In study 1, the following parameters were used: matrix size 64×64 voxels, FoV = 210 mm, 30 slices (ascending), slice thickness 4.5 mm (10% gap), TR = 1600 ms, TE = 30 ms, flip angle 90°. For the “dots-in-space” task (study 1), we used slightly different parameters (FoV = 192 mm, 35 slices (ascending), slice thickness 4 mm (10% gap), TR = 2150 ms). In total, 208 functional images were collected during the “dots-in-space” task, 215 scans for the Landmark task (version A) and 569 images for the mental rotation task. The initial images were excluded from further analyses in order to remove the influence of T1 stabilization effects.

In study 2, we aimed to optimize the acquisition sequence for the Landmark task (version B and C) in order to boost the relatively low t-statistics observed in study 1. Specifically, we used a sequence that had previously been shown to provide high BOLD sensitivity and–more importantly for the goal of study 2 –excellent test-retest reliability of BOLD activation for a face perception paradigm [17]. The following scanning parameters were used: matrix size 64×64 voxels, FoV = 192 mm, 30 slices (descending), slice thickness 4 mm (15% gap), TR = 1450 ms, TE = 25 ms, flip angle 90°. In total, 222 functional images were collected for each subject.

### 2.4 MRI data analysis

All fMRI data were analyzed using the standard routines and templates from the software package SPM8 (v4290; www.fil.ion.ucl.ac.uk/spm) in MATLAB 7.7.0.471 (R2008b) (The MathWorks, Inc.). Functional images were realigned, normalized (using the standard SPM EPI-Template), resampled to a voxel size of 2×2×2 mm^3^, smoothed with a 5-mm isotropic Gaussian kernel, and high-pass filtered (cut-off period 128 s). After pre-processing, statistical analysis was performed in a two-stage, mixed-effects procedure. At the single-subject level, BOLD responses were modelled in a General Linear Model (GLM) using boxcar functions convolved with the canonical hemodynamic response function from SPM8 [32,33]. For the “dots-in-space” task, we modelled four conditions (i.e., control, easy, hard, and baseline; instructions were not modelled). For the mental rotation task, we modelled three conditions (i.e., high spatial processing load, low spatial processing load, and fixation baseline; instructions were not modelled). For the Landmark task, we modelled two conditions (i.e., activation and control; instructions were not modelled). Additionally, the six realignment parameters were included as nuisance regressors in each design matrix to control for movement-related artifacts. For each paradigm and subject, contrast images were computed by contrasting activation and control conditions. More specifically, the following linear contrasts were calculated for each subject: “dots-in-space” task: “easy + hard > 2*control”; mental rotation task: “high spatial processing load > low spatial processing load”; Landmark task: “activation > control”. At the group level, individual contrast images for each paradigm were entered into separate one-sample t-tests. The anatomical localization of activated brain regions was assessed both by the SPM anatomy toolbox [34] and the WFU-Pickatlas [35].

#### 2.4.1 Study 1: Comparison of imaging paradigms

In study 1, we tested whether the three visuospatial processing paradigms were able to robustly determine right-hemispheric dominance not only at the group level, but also at the individual-subject level. Here, we defined four (subjective) criteria for characterizing robustness of right-hemispheric activation. These criteria assessed whether the paradigm activated a right-lateralized network both at the group level in a typical subject population (criterion a and b) and in a certain number of subjects at the single-subject level (criterion c and d):

a At the group level, the paradigm had to induce brain activity in a fronto-parietal network typically associated with spatial processing [11,30] at a significance level p < 0.001, cluster threshold k = 20.
b At the group level, the paradigm had to evoke right-hemispheric lateralization of brain activity, as indicated by a lateralization index LI < -0.4 [36], in core regions of the above-mentioned network (i.e., in frontal or parietal regions-of-interest (ROIs)).
c At the single-subject level, the paradigm had to induce brain activity in the right-hemispheric frontal and parietal ROIs at the significance level p < 0.001 uncorrected, cluster threshold k = 20, in more than 30% of all subjects.
d At the single-subject level, at least 30% of the subjects had to show right-hemispheric lateralization of the brain activation pattern (LI < -0.4) in both the frontal and parietal ROI.

According to our previous experience with functional imaging tasks assessing spatial processing (e.g.,[29,30,37]) we expected that the strength of activity (in terms of t-values) would be rather low at the individual subject level. We therefore opted at this point for a liberal whole-brain threshold for the single-subject analyses (i.e., 30%) to not exclude paradigms that might provide reliable but weak (in terms of t-values) measures of hemispheric dominance in some subjects.

Analysis 1: Brain activation at the group level: Brain activation patterns for each paradigm were first analyzed at the group level. One-sample t-tests were calculated separately for each paradigm, based on the contrast images from the single-subject analysis.

ROI masks definition: ROI masks were defined using an approach based on Jansen et al. 2006 [38]. As visuospatial processing is subserved by a large neurocognitive network, with different regions within this network showing different extents of lateralization, the LI is unlikely to reveal a consistent pattern of lateralization across these key regions of the brain. Therefore, constructing precise ROIs that capture the brain activity is of great importance. For this, two approaches, with their own advantages and disadvantages, are commonly used: ROIs can be defined either functionally, based on the pattern of activation, or anatomically, based on anatomic knowledge. Obvious disadvantages of the latter are that pure anatomical definitions might include areas that are not engaged by the task or, vice versa, might exclude activation of interest that lies outside the chosen ROI (e.g., due to inaccuracies in the normalization procedure). Additionally, macroscopic landmarks are rarely reliable indicators of cytoarchitectonic borders [39]. On the other hand, functionally defined ROIs are also subject to limitations as they are typically derived from data of a pilot study investigating the functional activation in another group of subjects or within the same cohort with similar paradigms [40,41]. Consequently, they might include regions outside the actual area of interest–thus, often necessitating additional masking.

To accommodate for the respective weaknesses of anatomically and functionally defined ROIs, we here applied a combined approach, which is based on both anatomical and functional constraints, for the definition of frontal and parietal ROIs as described in Adcock et al. (2003) [40] and Jansen et al. (2004) [37]:

For the frontal ROI, group level activation patterns were thresholded as follows: “dots-in-space” and mental rotation task with p < 0.001 uncorrected, k = 50; Landmark task version A, B and C with p < 0.01 uncorrected, k = 50 (version A) and k = 20 (Version B and C), reflecting the functional constraint. We then applied anatomical constraints based on prior anatomical knowledge. Specifically, we used the frontal lobe mask as given by the WFU-Pickatlas as an inclusive mask to differentiate between regions of interest and regions of no interest. For each paradigm, we then created the frontal mask as a combination of the activated voxels surviving the defined thresholds within the anatomical landmarks.”

**Fig 3:**
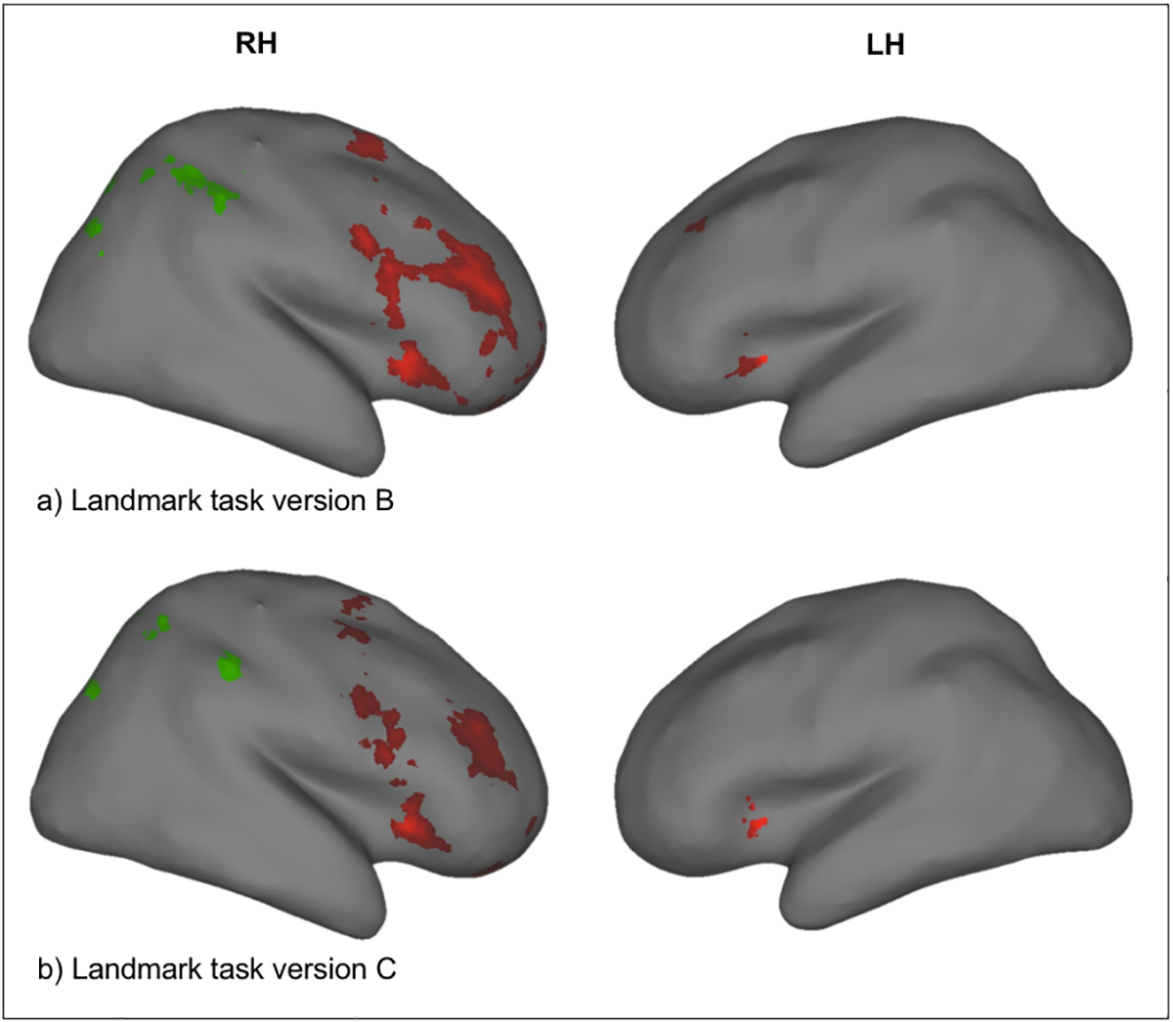
Landmark task ROIs. Frontal (red) and parietal (green) ROIs are shown for Landmark task version B (a) and C (b). Frontal and parietal ROIs were defined by creating a mask, resembling the conjunction of the group level activation patterns of session 1 and 2 within the frontal lobe and parietal lobe, respectively, as given by the WFU-Pickatlas (p < 0.01 uncorrected, k = 20). RH = right hemisphere, LH = left hemisphere.

For the parietal ROI, the same procedure as for the frontal ROI was used. For each paradigm, parietal ROIs were defined by creating masks of the group level activation pattern (“dots-in-space” and mental rotation task with p < 0.001 uncorrected, k = 50; Landmark task version A with p < 0.001, k = 100; Landmark task version B and C with p < 0.01, k = 20) within the anatomical constraints of the parietal lobe as given by the WFU-Pickatlas. The specific thresholds were chosen to ensure that masks were roughly similar in size for the different paradigms. Note that, in study 2, the conjunction of the group level activation patterns of session 1 and 2 was used to define frontal and parietal ROIs (Fig 3). In total, we created 10 masks–that is, a frontal and a parietal one for each paradigm.

The degree of hemispheric lateralization was quantified by the lateralization index (LI), which is given by the formula

$$LI=\frac{A_{L}-A_{R}}{A_{L}+A_{R}},$$

where A_L_ and A_R_ refer to measures of fMRI-activity for equal ROIs within the left (L) and right (R) hemisphere, respectively. Several approaches have been established to calculate the LI (for a discussion, see [14]). We here applied the bootstrapping approach implemented in the SPM8 LI-toolbox, which is the current gold standard [36]. The bootstrap approach uses 20 thresholding intervals with equally sized steps from 0 to the maximum t-value in the investigated region. At each threshold 100 bootstrap, resamples with a resample ratio of k = 0.25 were generated for each side from all the voxels in the investigated ROIs. From these resamples, all 10,000 possible LI combinations were calculated. A trimmed mean is then computed by only considering the central 50% of data points to exclude statistical or artefactual outliers and, thus, enhance the stability of the estimate. In a last step, a weighted mean LI is calculated by weighting the LIs with the respective thresholds, with higher thresholds receiving higher weights. The LI values range from −1 to +1. Positive values indicate a left-hemispheric dominance and negative values indicate a right-hemispheric dominance. We masked out the midline (+/- 5 mm) to avoid flow artifacts in the large draining veins, as proposed by Wilke and Schmithorst [36]. LIs for the group level BOLD patterns were calculated from the activation in the defined frontal and parietal masks for each paradigm.

Analysis 2: Brain activation in single subjects: The ROIs resulting from each paradigm’s group analysis were used to investigate the activation strength in individual subjects. Therefore, single-subject activation maps were screened for activation in the respective ROIs at a fixed significance threshold (p < 0.001 uncorrected, cluster threshold k = 20). Individual LIs were also calculated in these ROIs, resulting in two indices (one frontal and one parietal LI) per subject for each paradigm.

#### 2.4.2 Study 2: Test-retest reliability

In study 2, we assessed the test-retest reliability of both the activation patterns and the lateralization of the spatial processing network. Notably, we restricted our analyses to the Landmark task since this was the only task that fulfilled all criteria of robustness described above (see Results). First, we quantified the reliability of the activation patterns by computing intra-class correlation coefficients (ICCs) for each voxel using the ICC toolbox extension within SPM [42]. We then assessed the reliability of the brain lateralization of the spatial attention network. As a measure of the test-retest reliability of the *degree of lateralization*, we computed an ICC (two-way mixed model with absolute agreement using SPSS; IBM SPSS Statistics for Macintosh, version 22.0) for the LIs in the frontal and parietal ROI, respectively. As a measure of the test-retest reliability of *hemispheric dominance* (i.e., left, right), we determined the percentage of subjects in which categorical decision on the dominant hemisphere was consistent across measurements. Since the exact thresholds for partitioning left-dominance, right-dominance and bilateral activation are somewhat arbitrary, we repeated our analyses for three different specifications to account for this issue:

(i) Three categories; left dominance for LI>0.4, right dominance for LI < -0.4, bilateral activation for |LI| ≤ 0.4
(ii) Three categories; left dominance for LI > 0.2, right dominance for LI < -0.2, bilateral activation for |LI| ≤ 0.2
(iii) Two categories; left dominance for LI > 0, right dominance for LI ≤ 0

## 3. Results

### 3.1. Study 1: Comparison of imaging paradigms

**Fig 4:**
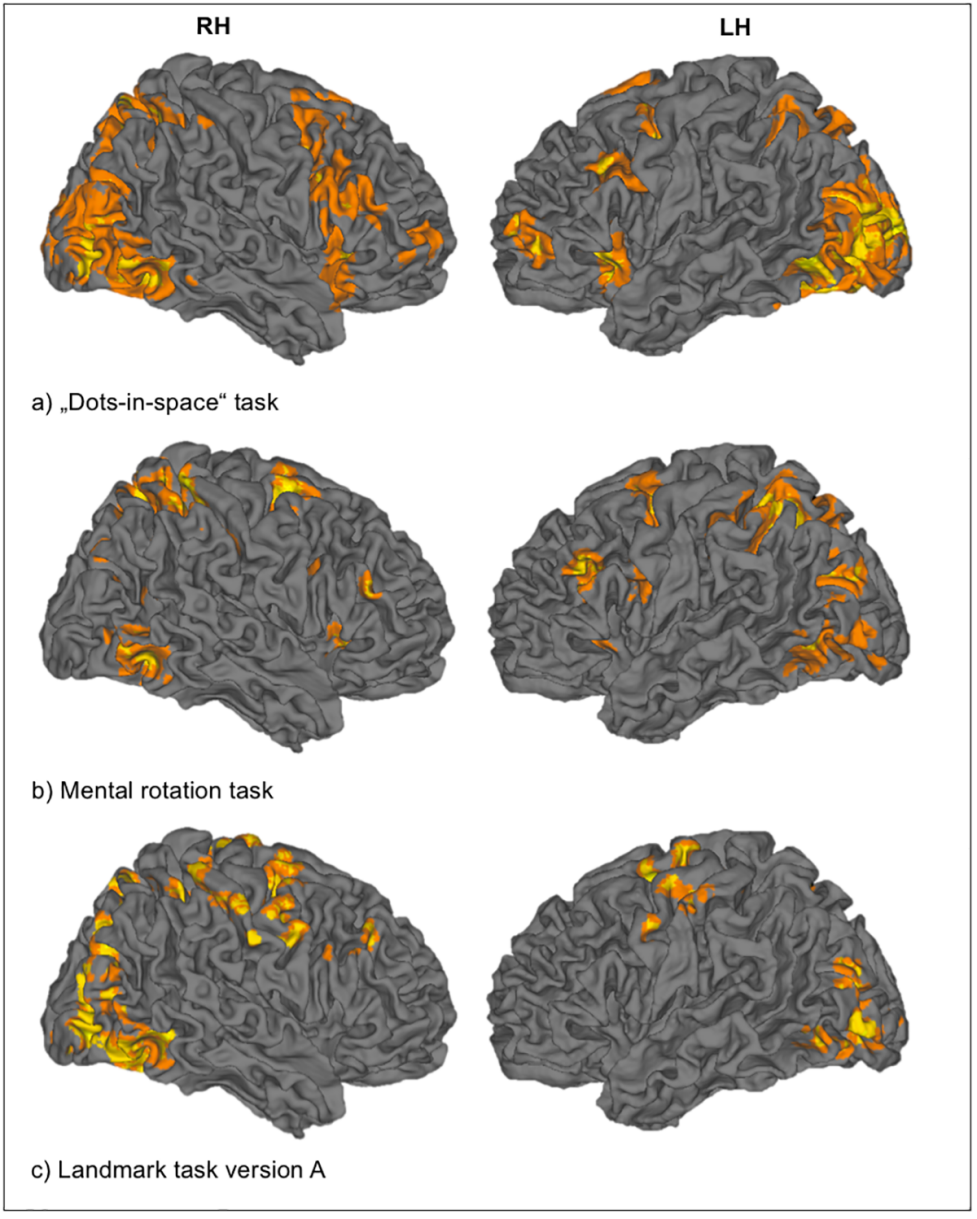
Group activation patterns. Group activation patterns evoked by the “dots-in-space” task, the mental rotation task and the Landmark task version A (p < 0.001 uncorrected, k = 20). RH = right hemisphere, LH = left hemisphere. A detailed overview of the activated brain areas can be found in the Supplementary section (S1).

“Dots-in-space” task: Behavioral data: Mean hit rate in the easy condition was 73.0 ± 14.4%, in the difficult condition 63.0 ± 17.0%, and in the control condition 98.5 ± 2.5%. A one-way ANOVA for repeated measures revealed an expected, significant main effect of difficulty (p < 0.001) across conditions. Imaging data: At the group level, brain activity for the linear contrast “easy + difficult > 2*control” (p < 0.001 uncorrected, k = 20) was found in a fronto-parietal network, in the anterior cingulate cortex and in the occipital lobe (Fig 4A, Table 1). At the group level, brain activity in the parietal cortex was right-lateralized (LI = -0.42) and bilateral to right-lateralized in the frontal cortex (LI = -0.24). At the single-subject level, brain activity at p < 0.001 uncorrected, k = 20, was found in 15/15 subjects in the right frontal ROI and in the right parietal ROI. Brain activity was right-lateralized (LI < -0.4) for 7/15 subjects in the frontal ROI and for 6/15 subjects in the parietal ROI. However, only three subjects showed right-lateralized activation when looking at both ROIs simultaneously, whereas the remaining subjects showed bilateral LIs in either the frontal and/or parietal ROIs.

**Table 1:** Study participants’ characteristics, study 1. Age, sex and the lateralization indies for the”dots-in-space” (Dis), the mental rotation (MR) and the Landmark task version A (Lt). The additional column shows the number of voxel surviving the threshold (p<0.001 uncorrected, k = 20) in the respective right-hemispheric ROI.

Subject ID	age	sex	LI Dis (frontal ROI)	active voxel (p < .001 unc.; k = 20)	LI Dis (parietal ROI)	active voxel (p < .001 unc.; k = 20)	LI MR (frontal ROI)	active voxel (p < .001 unc.; k = 20)	LI MR (parietal ROI)	active voxel (p < .001 unc.; k = 20)	LI Lt (frontal ROI)	active voxel (p < .001 unc.; k = 20)	LI Lt (parietal ROI)	active voxel (p < .001 unc.; k = 20)
**1.1**	24	f	0,10	1167	-0,35	1254	0,90	148	0,82	25	-0,60	455	-0,47	205
**1.2**	25	f	0,05	157	-0,37	346	0,68	443	0,89	-	0,59	-	-0,17	-
**1.3**	20	f	-0,39	1687	-0,53	895	0,67	442	-0,06	607	-0,38	20	0,28	-
**1.4**	24	m	-0,42	1300	-0,46	1403	0,49	1121	0,58	915	-0,46	61	-0,50	60
**1.5**	21	f	-0,16	1236	-0,12	2098	0,57	1280	0,65	935	-0,44	-	-0,73	-
**1.6**	26	f	-0,43	1171	-0,31	1916	0,25	187	0,49	969	0,17	-	-0,44	92
**1.7**	26	m	-0,57	1189	-0,30	902	0,57	925	0,58	690	0,45	-	0,55	-
**1.8**	25	m	0,34	62	-0,21	117	0,77	-	0,31	50	-0,41	-	0,22	-
**1.9**	23	f	-0,47	57	0,07	1105	0,66	796	0,55	801	-0,22	-	-0,49	61
**1.10**	32	m	-0,84	547	-0,71	348	-0,44	1228	-0,78	1032	-0,06	160	0,29	62
**1.11**	25	m	0,13	166	-0,41	514	0,66	948	0,70	926	0,00	-	-0,13	-
**1.12**	24	f	-0,31	2103	-0,48	2402	-0,07	808	-0,06	1066	-0,65	284	-0,54	420
**1.13**	24	f	-0,64	801	-0,11	532	0,57	907	0,53	763	-0,34	162	-0,56	363
**1.14**	26	m	0,23	161	-0,28	679	0,62	1487	0,51	1495	-0,39	-	-0,67	-
**1.15**	24	f	-0,55	170	-0,73	516	0,22	381	0,01	388	-0,63	-	-0,86	-

(m = male; f = female; LI = lateralization index; ROI = region of interest)

As suggested by one of our reviewers, we next performed correlation analyses between performance levels (i.e., hit rates) and measures of BOLD activation and hemispheric lateralization in order to address whether the observed inter-individual differences in brain activity and lateralization were related to the behavior (i.e., task performance) of individual subjects. We found a positive correlation between performance levels of the “dots in space” task and the number of activated voxels (at a statistical threshold of p<0.001 uncorrected, k = 20, in the right frontal ROI (Spearman Rho correlation coefficient: *ρ* = 0.528 (p = 0.043) and *ρ* = 0.559 (p = 0.030) for the easy and hard condition of the task, respectively). On the contrary, no significant correlations were found for the right parietal ROI (*ρ* = 0.455 (p = 0.088) and *ρ* = 0.361 (p = 0.186) for the easy and hard condition, respectively). Additionally, we tested whether for a relation between task performance and lateralization strength of BOLD activations by computing correlations between the hit rates of the easy and hard condition and the LIs of frontal and parietal ROIs. However, no significant correlations between these variables were observed (see S2 Table).

Mental rotation task: Behavioral data: Mean hit rate in the easy condition was 97.2 ± 7.3%, in the difficult conditions 16.2 ± 10.2%, thus showing the expected effect of difficulty across conditions (p < 0.001), but also pointing to the extremely challenging nature of the activation condition. Subjects were essentially performing at chance with no significant difference from chance level (20%, p = 0.168). Note that, despite the low hit rate in the difficult condition, subjects reported to have actively engaged in the task rather than purely guessed the orientation of the cubes, as assessed by debriefing after the experiment. Imaging data: At the group level, brain activity for the linear contrast “high spatial processing load > low spatial processing load” (p < 0.001 uncorrected, k = 20) was found in a fronto-parietal network and in the anterior cingulate cortex (Fig 4B, Table 1). However, in contrast to our expectations, the brain activity was left-lateralized in both the parietal (LI = 0.44) and the frontal cortex (LI = 0.60). At the single-subject level, brain activity at p < 0.001 uncorrected, k = 20, was found in 14/15 subjects in frontal and parietal ROIs in both hemispheres. Brain activity was right-lateralized (LI < -0.4) for only one subject in both the frontal and parietal ROI, but left-lateralized in both ROIs in nine subjects. We again tested for a brain-behavior interaction by computing correlations between the task performance and measures of brain activity. No significant correlation between performance levels (i.e., hit rates) of the task and the number of activated voxels (at a statistical threshold of p<0.001 uncorrected, k = 20) in the right frontal (*ρ* = 0.468; p = 0.092) or right parietal ROI (*ρ* = 0.257; p = 0.376) were detected. Similarly, no significant correlations were observed between task performance and the LIs (see S2 Table).

Landmark task (version A): Behavioral data: Mean hit rate in the activation condition was 86.1 ± 15.9% and in the control condition 87.3 ± 17.8%. Hence, in contrast to the “dots-in-space” task and the mental rotation task, we did not observe a significant difference in the hit rate between activation and control condition (p = 0.781). Imaging data: At the group level, brain activity for the linear contrast “activation > control” (p < 0.001 uncorrected, k = 20) was found in a fronto-parietal network and the anterior cingulate cortex (Fig 4C, Table 1). Brain activity was right-lateralized in the parietal cortex (LI = -0.63) and bilateral in the frontal cortex (LI = 0.03). At the single-subject level, brain activity at p < 0.001 uncorrected, k = 20, was found in 6/15 subjects in the right frontal ROI and in 7/15 subjects in the right parietal ROI. Furthermore, 5/15 subjects showed brain activity in both regions in the right hemisphere. Brain activity was right-lateralized (LI < -0.4) for 6/15 subjects in the frontal ROI (two subjects showed left-lateralized activation with LI > 0.4) and for 9/15 subjects in the parietal ROI (one subject showed left-lateralized activation with LI > 0.4). Five subjects displayed right-lateralized activation in both ROIs and one subject was left-lateralized in the frontal and parietal ROI. As for the other two tasks, we assessed correlations between the task performance and measures of brain activity in order to test for brain-behavior interactions. However, no significant correlations between performance levels (i.e., hit rates) of the Landmark task and the number of activated voxels (at a statistical threshold of p<0.001 uncorrected, k = 20) in the right frontal (*ρ* = -0.213; p = 0.686) or right parietal ROI (*ρ* = -0.273; p = 0.554) were detected. Similarly, no significant correlations were found between task performance and the LIs (see S2 Table).

#### 3.1.1 Evaluation of paradigms

All tasks showed, at the group level, brain activity in a fronto-parietal network related to spatial processing (criterion a). This is in line with the previous studies that had utilized the three tested paradigms [12, 27, 28]. This activity was right-lateralized for the Landmark task and the “dots-in-space” task, but not for the mental rotation task (criterion b). Given that we aimed to establish a paradigm that induces right-hemispheric lateralization, the latter task did not fulfill one important requirement. At the single-subject level, all three paradigms fulfilled the criterion of “robust activation in core-regions” (criterion c). For each task, we found activity in the frontal and parietal cortex (at least in the dominant hemisphere) at a specified threshold (p < 0.001 uncorrected). Furthermore, right-lateralized brain activity at the single-subject level in the frontal or parietal cortex, or both, was observed for the “dots-in-space” task and the Landmark task in a subset of the subjects. This suggests that both tasks are, in principle, suited for studying right-hemispheric lateralization. However, the Landmark task yielded slightly higher ratios and was the only paradigm that fulfilled criterion d (but note, this wouldn’t have been the case when using a slightly more conservative threshold, e.g., > 40%). Furthermore, it was the most time-efficient paradigm in the sense that it provided equally (or even slightly more) robust results, while lasting only 5 minutes (as compared to 9 minutes for the “dots-in-space” task). Hence, we chose the Landmark task as the most promising paradigm for efficiently and robustly assessing right-hemispheric dominance related to spatial processing in single subjects. Note that this was also in line with the current view, suggesting the Landmark task to be the state-of-the-art paradigm for inducing right-hemispheric lateralization. A summary of the results can be found in Table 1.

Critically, although inducing right-hemispheric activation most consistently across the tested paradigms, the Landmark task in its present form showed somewhat weak overall activation strength (as illustrated by the fact that only 5/15 subjects showed significant activation in both ROIs at the pre-specified threshold). This represents a limiting factor when being interested in single-subject effects. For study 2, which addressed the test-retest-reliability of our selected (Landmark task) paradigm, we therefore aimed to induce stronger activations by optimizing the task and acquisition sequence (see Discussion).

### 3.2. Study 2: Test-retest reliability

In study 2, we assessed the test-retest reliability for two adapted versions of the Landmark task (version B and C). Consistent with study 1, we here first describe the behavioral results, followed by the characteristics of the activation patterns at the group and single-subject level for both versions of the Landmark task (3.2.1). We then report the results of the reliability analyses (3.2.2).

#### 3.2.1 Comparison of both versions of the Landmark task

**Fig 5:**
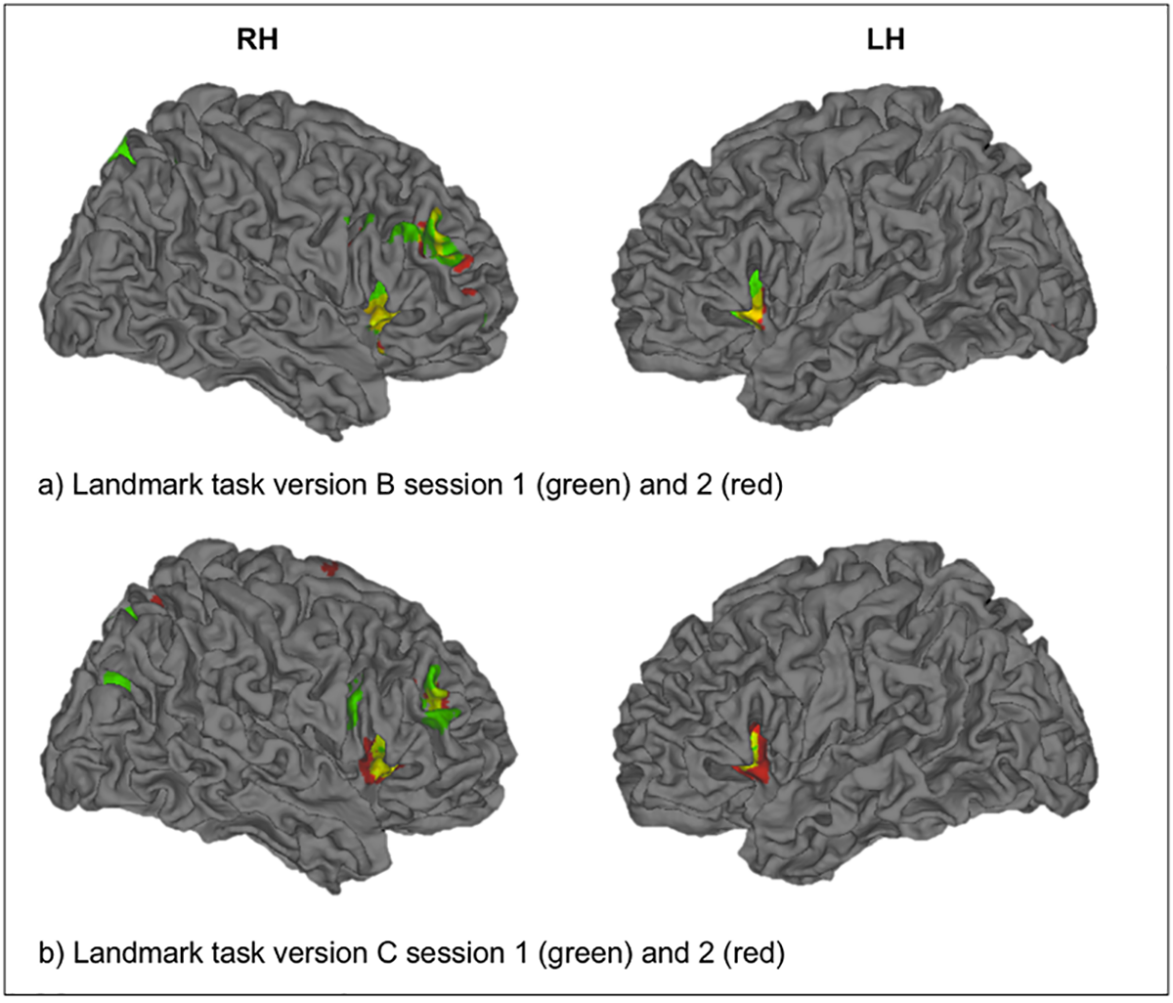
Group activation patterns evoked by the Landmark task version B and C. Activation of the first (green) and second session (red) are plotted together (p < 0.001 uncorrected, k = 20) for version B (top) and version C (bottom), respectively. Yellow colored regions indicate voxels that were active in both sessions. RH = right hemisphere, LH = left hemisphere.

Landmark task (version B): Behavioral data: Mean hit rate in the activation condition was 75.8 ± 10.9% in session 1 and 78.1 ± 9.1% in session 2. Mean hit rate in the control condition was 84.6 ± 12.3% in session 1 and 90.9 ± 8.4% in session 2. Hit rates in the activation and control conditions were significantly different in both sessions (p < 0.001), suggesting that the revised version of the Landmark task indeed induced performance differences. Imaging data: At the group level, brain activity for the linear contrast “activation > control” was found in a fronto-parietal network and the anterior cingulate cortex (Fig 5A, Table 2). At the group level, brain activity was right-lateralized both in the parietal cortex (session 1: LI = -0.88, session 2: LI = -0.86) and in the frontal cortex (session 1: LI = -0.85, session 2: LI = -0.90). At the single-subject level, brain activity at p < 0.001 uncorrected, k = 20, in the right frontal ROI was found in 13/20 subjects in session 1 and in 16/20 subjects in session 2. In the right parietal ROI, we found brain activity in 10/20 subjects in session 1 and in 10/20 subjects in session 2. For both session 1 and 2, 9/20 subjects showed brain activity in both regions. Brain activity was right-lateralized (LI < -0.4) for 16/20 subjects in session 1 and for 17/20 subjects in session 2 in the frontal ROI. For the parietal ROI, 14/20 subjects were right-lateralized for both session 1 and 2. 12 subjects displayed right-lateralized activation in both ROIs for both session 1 and 2, and only one subject was left-lateralized in the frontal and parietal ROI (see Table 2) in session 1. Hence, for this version of the Landmark task, right-hemispheric lateralization at the single-subject level could be detected in the majority of people in both ROIs (60%), and in almost all people in at least one of the regions (85% for session 2). This represents a notable improvement in robustness as compared with the initial version (A) of the Landmark task due to the optimized task and acquisition sequence.

**Table 2:** Study participants’ characteristics, study 2 and lateralization indices for the Landmark Task version B (LtB). The additional column shows the number of active voxel surviving the threshold (p<0.001 uncorrected, k = 20) in the respective right-hemispheric ROI.

Subject ID	age	sex	LI LtB (frontal ROI) Session I	LI LtB (frontal ROI) Session II	active voxel (p < .001 unc.; k = 20) session I	active voxel (p < .001 unc.; k = 20) session II	LI LtB (parietal ROI) Session I	LI LtB (parietal ROI) Session II	active voxel (p < .001 unc.; k = 20) session I	active voxel (p < .001 unc.; k = 20) session II
**2.1**	27	f	-0,89	-0,62	646	664	-0,49	-0,51	125	115
**2.2**	25	m	-0,64	-0,88	-	289	-0,84	-0,40	-	71
**2.3**	24	m	-0,31	-0,72	688	45	-0,69	-0,59	251	-
**2.4**	30	f	-0,85	-0,82	110	1465	-0,93	-0,74	-	228
**2.5**	25	m	-0,79	-0,68	257	-	-0,65	-	-	-
**2.6**	25	f	-0,61	-0,19	1156	76	-0,24	-0,81	244	-
**2.7**	24	f	-0,71	-0,75	399	1177	-0,50	-0,45	34	233
**2.8**	25	f	-0,85	-0,80	1266	-	-0,89	-	29	-
**2.9**	24	f	-0,71	-0,78	1227	512	-0,02	0,01	101	153
**2.10**	21	f	-0,80	-0,71	357	1263	-0,45	-0,13	33	179
**2.11**	25	f	-0,88	-0,72	-	449	-0,08	-0,64	-	30
**2.12**	22	m	-0,03	-0,64	-	-	-0,13	-0,63	-	29
**2.13**	29	m	-0,42	-0,48	1505	305	-0,42	-0,44	106	-
**2.14**	25	f	0,55	-0,02	52	23	0,50	-	-	-
**2.15**	23	m	-0,81	-0,53	54	46	-0,77	-0,54	-	-
**2.16**	27	m	-0,70	-0,81	192	412	-0,77	-0,94	26	-
**2.17**	26	m	-0,82	-0,85	-	-	-0,82	-0,91	-	-
**2.18**	27	f	-0,88	-0,16	-	105	-	-0,82	-	34
**2.19**	22	m	-0,22	-0,82	-	140	-0,69	-0,37	-	-
**2.20**	24	m	-0,83	-0,60	-	640	-0,55	-0,68	71	149

Landmark task (version C): Behavioral data: Mean hit rate in the activation condition was 72.2 ± 8.5% in session 1 and 76.2 ± 9.5% in session 2. Mean hit rate in the control condition was 82.2 ± 23.1% in session 1 and 90.6 ± 11.5% in session 2. Activation and control conditions were significantly different in session 1 (p = 0.048) and session 2 (p < 0.001), again suggesting that the revised version induced performance differences. Imaging data: At the group level, brain activity for the contrast “activation > control” was found in a fronto-parietal network and the anterior cingulate cortex (Fig 5B, Table 3). Brain activity was right-lateralized both in the parietal cortex (session 1: LI = -0.83, session 2: LI = -0.74) and in the frontal cortex (session 1: LI = -0.80, session 2: LI = -0.90). At the single-subject level, brain activity at p < 0.001 uncorrected, k = 20, in the right frontal ROI was found in 15/20 subjects in session 1 and in 13/20 subjects in session 2. For the parietal ROI, 8/20 subjects showed activation in session 1 and 10/20 subjects in session 2. Furthermore, 7/20 subjects showed brain activity in both ROIs in session 1 and 10/20 subjects in session 2. Brain activity in the frontal ROI was right-lateralized (LI < -0.4) for 15/20 subjects in session 1 and in session 2. For the parietal ROI, brain activity was right-lateralized for 10/20 subjects in both sessions. Additionally, 9/20 subjects displayed right-lateralized activation for both ROIs in session 1 and 8/20 subjects in session 2. Only one subject was left-lateralized in the frontal and parietal ROI (see Table 3) in session 2.

**Table 3:** Lateralization indices for the Landmark task version C (LtC). The additional column shows the number of active voxel surviving the threshold (p<0.001 uncorrected, k = 20) in the respective right-hemispheric ROI.

Subject ID	LtC (frontal ROI) Session I	LtC (frontal ROI) Session II	active voxel (p < .001 unc.; k = 20) Session I	active voxel (p < .001 unc.; k = 20) Session II	LtC (parietal ROI) Session I	LtC (parietal ROI) Session II	active voxel (p < .001 unc.; k = 20) session I	active voxel (p < .001 unc.; k = 20) session II
**2.1**	-0,77	-0,76	61	503	-0,19	-0,84	46	120
**2.2**	-0,84	-0,91	25	-	-0,35	-0,06	-	-
**2.3**	-0,40	-0,73	-	163	-0,82	-0,65	33	-
**2.4**	-0,82	-0,73	1132	373	-0,69	-0,1	198	110
**2.5**	-0,82	-0,58	32	-	-0,69	-0,35	-	-
**2.6**	-0,59	-0,04	178	96	-0,88	-0,36	-	-
**2.7**	-0,75	-0,75	25	529	-0,21	-0,26	-	72
**2.8**	-0,87	-0,77	290	655	-0,99	-0,68	-	177
**2.9**	-0,69	-0,80	580	626	0,03	-0,03	295	98
**2.10**	-0,59	-0,79	479	65	0,26	-0,55	72	-
**2.11**	-0,55	-0,76	183	-	-0,58	-0,49	62	-
**2.12**	-0,48	-0,08	-	-	-0,92	0,50	-	-
**2.13**	-0,34	-0,17	334	268	-0,22	-0,69	-	133
**2.14**	0,60	0,40	58	876	0,71	0,64	-	85
**2.15**	-0,67	-0,52	23	-	-0,73	-	-	-
**2.16**	-0,28	-0,69	-	-	-	-0,64	-	-
**2.17**	-0,85	-0,76	-	506	-0,29	-0,85	-	88
**2.18**	0,01	-0,41	1370	584	-0,56	-0,58	322	101
**2.19**	-0,32	-0,67	453	-	0,07	-0,1	72	-
**2.20**	-0,51	0,05	-	40	-0,41	-0,81	-	52

#### 3.2.2 Test-Retest Reliability

Test Retest Reliability was assessed in a four-step procedure: While the first two analyses steps were performed with regard to the whole brain BOLD activation patterns, the third and fourth analysis step concerned the test-retest reliability of the obtained LIs:

1. Qualitative analysis of the group activation overlap
2. Test-retest reliability of voxelwise activation strength across subjects
3. Test-retest reliability of the degree of lateralization
4. Analysis of consistency of the categorical classification of hemispheric dominance

We first provide a qualitative analysis of consistency of activation patterns across the different measurements by inspecting the overlap of brain activation patterns at the group level. These overlaps show that for both versions (i.e., B and C) of the Landmark task, a comparable frontal network was activated in session 1 and session 2 (Fig 5).

**Fig 6:**
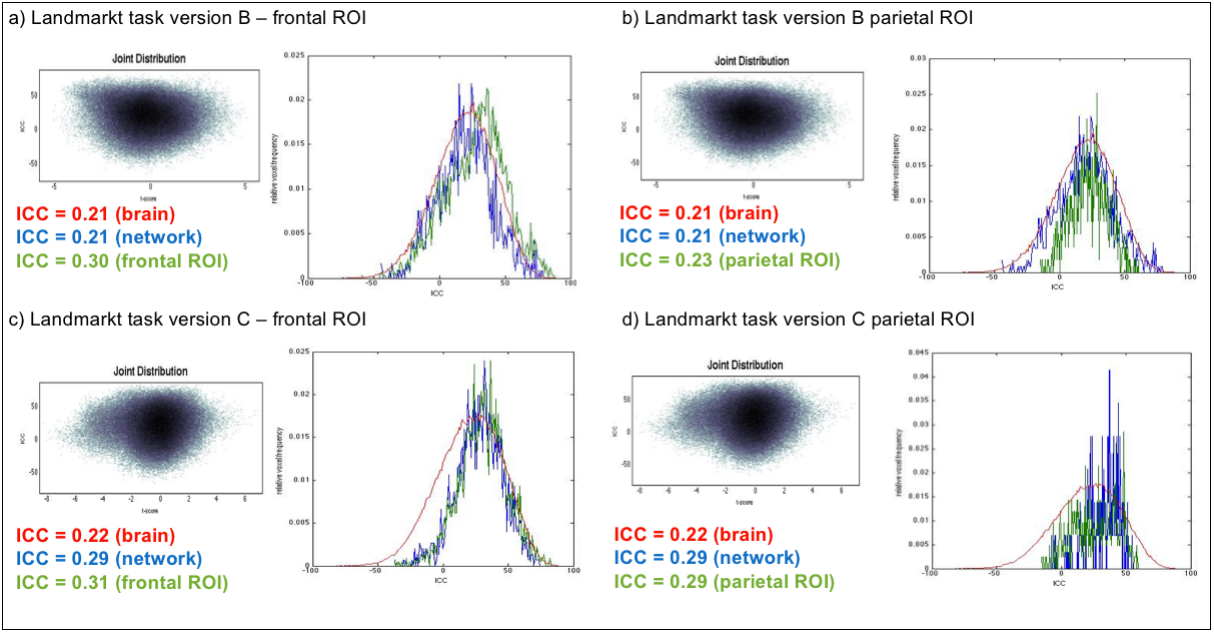
ICC results. Left (top and bottom): Joint probability distribution of voxel-wise t-values and associated median ICC values. Right (top and bottom): ICC frequency distributions for the whole brain (red), for the voxels in the activated network (blue) and for the defined frontal and parietal ROI (green). The “activated network” was defined based on the results from the first measurement. Voxels were classified as active if they had t-values t>3.5794 (corresponding to p < 0.001 uncorrected, k = 20). Both diagrams are presented for the Landmark tasks version B (a, b) and C (c, d), for the frontal and the parietal ROI, respectively.

Second, with regard to the test-retest reliability of the activation patterns, we computed ICCs for each voxel using the ICC toolbox extension within SPM (Caceres et al., 2009). Whole-brain joint probability distributions showed an association between t-values and ICCs (Fig 6). According to established conventions, we classified test-retest reliability as “poor” for ICC ≤ 0.4, “fair” for 0.4 < ICC ≤ 0.6, “good” for 0.6 < ICC ≤ 0.8, and “excellent” for ICC > 0.8 [42,43]. For both versions of the paradigm, ICCs were higher for voxels showing strong activation (high t-values) or “deactivation” (high t-values for the opposite contrast). For the Landmark task version B, the median ICC for the whole brain was 0.21, for the activated network 0.21, for the frontal ROI 0.30, and for the parietal ROI 0.23 (Fig 6A and 6B). For the Landmark task version C, the median ICC for the whole brain was 0.22, for the activated network 0.29, for the frontal ROI 0.31, and for the parietal ROI 0.29 (Fig 6C and 6D). All these values indicate poor reliability, suggesting that the BOLD signal in individual voxels was not very reliable.

Notably, however, the main goal of study 2 was to determine whether measures of hemispheric lateralization (i.e., degree of hemispheric lateralization and the categorical classification of hemispheric dominance) were reliable across multiple sessions. In a third step, we therefore focused on these quantities. With regard to the test-retest reliability of the degree of lateralization, the average measures for ICCs for the LIs in the frontal (ICC = 0.57) and in parietal ROI (ICC = 0.54) suggested fair reliability of this measure in Landmark task version B. In Landmark task version C, average measures for ICCs were excellent (ICC = 0.81) and poor to fair (ICC = 0.33) in the frontal and parietal ROI, respectively.

In a fourth step, we tested the consistency of the categorical classification of hemispheric dominance across both sessions. When using a conservative threshold of |LI| > 0.40, 70.0% of subjects showed consistently right-hemispheric dominance in the frontal and 62.5% in the parietal ROI (version B). For version C, these percentages were reduced, with 60.0% and 27.8% of subjects showing consistently right-hemispheric dominance in the frontal and parietal ROI, respectively. As mentioned above, the chosen threshold of |LI| > 0.40 is rather conservative and, in fact, various studies in the literature have used more liberal thresholds for identifying left- or right-hemispheric lateralization [37,44]. In a more exploratory analysis, we used a more liberal criterion for hemispheric dominance (i.e., |LI| > 0.2) and found that 80.0% of subject showed right-hemispheric dominance in the frontal and 75.0% in the parietal ROI (version B). Again, these values were reduced for the version C with right-hemispheric dominance in the frontal ROI (70.0%) and parietal ROI (55.6%). When further softening the criterion for hemispheric dominance by considering only two categories (i.e., left, right), 95.0% of subjects showed right-hemispheric dominance in the frontal and 93.8% in the parietal ROI across both sessions in Landmark task version B. This percentage was marginally smaller (85.0% in the frontal, 72.2% in the parietal ROI) in Landmark task version C. These results again highlight to utility of the revised versions of the Landmark task for studying right-hemispheric lateralization using fMRI–at least for the liberal scenario when making binary decisions between left- or right-hemispheric dominance of the brain activation pattern.

## 4. Discussion

In the present study, we aimed to establish an fMRI paradigm that *robustly* and *reliably* evokes right-hemispheric lateralization of brain activity in the human brain. To this end, we first compared the suitability of three different paradigms on visuospatial processing: (i) the “dots-in-space” task (adapted from [28]), (ii) mental rotation task [24], and (iii) Landmark task [11]. These tasks had been used frequently in imaging studies to assess right-hemispheric lateralization in the human brain. In study 1, we evaluated the utility of these different paradigms to induce right-hemispheric lateralization in the spatial processing network. It is worth highlighting that, despite the fact that it is generally accepted that visuospatial processing induces right-hemispheric lateralization in the majority of subjects, studies have also reported notable variability across the population. Specifically, atypical (left-hemispheric) lateralization of visuospatial attention has also been described in a non-negligible proportion of subjects. Such inter-individual variability has not only been observed for visuospatial attention, but for any lateralized cognitive function, including language [5,7–9,45,46]. Hence, one would not expect that the tested visuospatial processing paradigms evoke right-hemispheric lateralization in each and every subject. This makes it somewhat challenging to devise a principled criterion to assess the “robustness” of the above-mentioned paradigms at the single-subject level. We have tried to accommodate for this inherent inter-individual variability by using rather liberal “robustness” criteria at the single-subject level in study 1.

Under these (subjective) criteria, we compared the “robustness” of the different paradigms. First, the mental rotation task failed, as it revealed a left-hemispheric lateralization of the activation pattern both at the group level, as well as in most of the individual subjects. Second, the “dots-in-space” task and the Landmark task performed more or less similar. More precisely, the Landmark task yielded slightly higher ratios of right-hemispheric dominance at the single-subject level than the “dots-in-space” task and passed all four criteria (but note that this would have not been the case for slightly more conservative criteria c and d). In an additional analysis, we asked whether the marked inter-subject variability observed in the brain activation patterns during all tasks was related to the behavior of individual subjects. While we found a significant correlation between performance levels (i.e., hit rates) and the number of activated voxels in the right frontal ROI for the “dots-in-space” task (at an uncorrected threshold not accounting for multiple comparisons), no other correlation reached significance. This suggests that a potential link between behavioral performance and the measures of brain activity tested in the present study was rather weak and thus could not sufficiently explain the variability in right-hemispheric lateralization observed across subjects. These differences are thus likely to relate to other factors such as distinct cognitive strategies [47], inter-subject variability in the BOLD signal [48–50], or variability in neuroanatomy [51].

Overall, we focused on the Landmark task (instead of the “dots-in-space” task) for study 2 because of its slight benefit in performance and the considerably higher efficiency (i.e., the Landmark task provided similarly robust results while taking only half the scan time). However, one has to carefully consider the limitations of the choice of fMRI paradigms for study 1. Here, we compared three commonly used paradigms, in a form in which they already existed in the literature (Landmark task and mental rotation task) or by closely adapting a paradigm designed for fTCD (“dots-in-space” task) for the use in fMRI. All three paradigms are however subject to several limitations (e.g., low performance in the mental rotation task, suboptimal implementation of the Landmark task in study 1), which might have confounded our selection of the most suitable and robust paradigm for study 2. In what follows, we highlight some of these issues to make the reader aware of the limitations of the present study.

With regard to the mental rotation task, subjects showed strikingly low behavioral performance levels during the activation condition, which aggravates the interpretation of the hemispheric lateralization results. The left-lateralized brain activity in the frontal and parietal ROIs is in contrast to the right-hemispheric lateralization reported by Dorst et al. [24] and the bilateral patterns observed by Hattemer and colleagues [26]. Several explanations might account for these differences. First, given the above-mentioned poor performance levels, we cannot exclude the possibility that subjects did not correctly engage in the mental rotation task (despite the fact that they reported active participation during the debriefing). To address this confound, forthcoming studies testing the utility of the mental rotation task should strive for a more graceful difficulty level to ensure adequate behavioral performance. Another potential source of variation between the present study and Dorst et al. [24] lies in the imaging method. While the present study used fMRI, Dorst and colleagues used fTCD. Notably, fTCD is sensitive primarily to major cerebral arteries, most commonly the middle cerebral arteries (MCA), which are insonated through the transtemporal window [52]. The MCA mainly supplies the lateral surface of the hemisphere with the exception of the superior parietal lobe, the inferior temporal lobe and the occipital lobe. Consequently, fTCD has relatively poor spatial resolution because signals can only be captured from brain regions that are supplied by the MCA. This results in rather crude estimates of hemispheric lateralization. Additionally, while fTCD might be suitable for studying brain areas completely supplied by the MCA or widely distributed network, brain regions lying outside the supply area of the MCA will not be captured at all. Given these marked differences between fTCD and fMRI, differences in the hemispheric lateralization results are to be expected to some degree–especially at the single-subject level where we observed high variability of the fronto- and parietal activation patterns.

An additional limitation of the present study is the small difference in the MRI acquisition parameters across the three paradigms (i.e., dots-in-space, mental rotation, and Landmark task). This might have resulted in somewhat different SNRs of the acquired data. Similarly, the differences in cognitive and behavioral characteristics of the tasks themselves (see Introduction) could have also biased our endeavor for identifying the most robust paradigm of right-hemispheric lateralization during visuospatial processing.

Finally, active and control conditions for each of the three visuospatial processing tasks differed in behavioral demands, as well as basic sensory and motor aspects, leading to differences between the two conditions arguably unrelated to the mechanisms underlying hemispheric lateralization.

Overall, these limitations suggest that future refinements of the utilized paradigms are needed for a more thorough investigation of the right-hemispheric lateralization in visuospatial attention. Having said this, the present study still makes a valuable contribution because it provides the (to our knowledge) first comparison of the robustness of currently established paradigms for assessing right-hemispheric dominance during visuospatial processing. As such, the present findings could serve as a benchmark against which future developments aiming to improve the robustness and reliability of “state-of-the-art” paradigms can be compared.

In study 2, we investigated the test-retest reliability of two (optimized) versions of the Landmark task. Test-retest reliability is an important test-theoretical property because, in fMRI, scientists are often confronted with poor SNR at the single-subject level [53] while, at the same time, being interested in inter-individual differences (e.g., in hemispheric lateralization) since they might offer deeper insights into the mechanisms underlying a cognitive task. Here, we find the reliability of the brain activation strength in single voxels to be poor, as indicated by low ICCs. This speaks to the above-mentioned poor SNR levels of the BOLD signal when only looking at single voxels individually. Having said this, the hemispheric dominance and the degree of hemispheric lateralization (as measured with the LI), which depicted the most important criteria in the present study, could be identified with reasonably high reliability across the two sessions. Specifically, we showed that for a binary classification of hemispheric dominance (i.e., left vs. right), right-hemispheric lateralization could be reliably detected across both sessions in the vast majority of subjects for the Landmark task version B (i.e., > 90% in the frontal and parietal ROI). Additionally, we found fair to good ICCs (ICC > 0.5) both in frontal and parietal ROIs for this version of the task. For version C, we report slightly lower reliabilities for the categorical classification of hemispheric dominance and the degree of hemispheric lateralization. We would like to stress out, that is a well-established procedure to study reliability when restricting the analysis to the activated brain network [37,40]. In this sense, our results can be interpreted as an upper bound on the reliability of the Landmark task, against which future studies that aim to refine and improve fMRI paradigms on visuospatial attention can be compared. Hence, our results provide evidence that the Landmark task version B should be preferred to reliably characterize hemispheric dominance.

In summary, comparing established paradigms for studying brain activity related to visuospatial processing, our results suggest that the Landmark task is best suited to assess right-hemispheric dominance of brain activation patterns and should be considered as the current method of choice for studying the lateralization of visuospatial attention with fMRI. This is supported by the reasonably high test-retest reliability of the LIs and the high consistency of hemispheric dominance classification across multiple sessions. Notably, these results are also in line with the current view that the Landmark task represents the state-of-the-art paradigm for investigating visuospatial functions, and is widely used both in clinical practice [11] and neuroscientific studies on hemispheric specialization [31]. Our results therefore suggest that the Landmark task, especially version B, robustly and reliably determines hemispheric dominance for visuospatial processing.

Establishing robust and reliable imaging paradigms to study hemispheric lateralization at the single-subject level will hopefully enable a more thorough understanding of the putative mechanisms [54]. In particular, developing precise models that capture which factors drive hemispheric specialization in individual subjects, how lateralization processes of different cognitive functions interact with each other, and how the brain integrates processes that are lateralized to opposite hemispheres. The relevance of such interactions among lateralized processes has been suggested for language and spatial attention [30,55,56], for language and working memory [57], and for face perception and handedness [17]. While moving in the right direction, these studies have not yet provided a principled and systematic investigation of the interactions among lateralized cognitive functions.

Along these lines, it is worth highlighting that the present study was part of a larger project that aims to establish a test battery for studying hemispheric lateralization of several cognitive functions (e.g., language, visuospatial processing, face processing and memory) and their interactions using fMRI. Specifically, this project strives to map how lateralized processes within one hemisphere interact (and compete) with each other, as well as the mechanisms by which the brain integrates processes lateralized to opposite hemispheres. Importantly, to ensure sensible inference on these mechanisms, the utilized paradigms need to adequately and reliably take into account the inter-individual variability in the degree of hemispheric lateralization mentioned above. The present study speaks to this endeavor of identifying such paradigms with respect to the right-hemispheric lateralization of visuospatial processing.

In summary, we here demonstrated the utility of the Landmark task for mapping right-hemispheric lateralization, but at the same time highlight current limitations of the paradigm. Further improvements of the present paradigm, as well as the use of more sophisticated scanner hardware and/or acquisition sequences (e.g., high field MRI, multiband EPI techniques), should constitute major endeavors of forthcoming studies in order to boost the sensitivity and stability of single-subject measures of hemispheric lateralization in the human brain.

## Supporting information

S1 Table Statistics of each paradigm’s group analysis. Detailed overview of the activated brain areas evoked by the functional paradigms.
(PDF)

S2 Table Non-significant correlations. Detailed overview of the non-significant correlations between task performance and the LIs.
(PDF)
