# Supplementary material for: Comparison of fMRI paradigms assessing visuospatial processing: Robustness and reproducibility
Source: PLoS One. 2017 Oct 23;12(10):e0186344. doi: 10.1371/journal.pone.0186344 (PMC5653292; doi:10.1371/journal.pone.0186344)
Supplement: S1 Table — Detailed overview of the activated brain areas evoked by the functional paradigms. (PDF) [file pone.0186344.s001.pdf]

S1: statistics of each paradigm’s group analysis

"Dots-in-space"task

Statistic: p-values adjusted for search volume

set-level		cluster-level				peak-level					
p	*c*	*p(FWE-corr)*	*p(FDR-corr)*	*kE*	*p(unc)*	*p(FWE-corr)*	*p(FDR-corr)*	*T*	*equivZ*	*p(unc)*	*x,y,z {mm}*
0,00115903	10	0	9,26E-101	23147	2,20E-102	1,25E-05	0,0014703	16,6488495	6,43058647	6,36E-11	[14;-90;14]
						4,24E-05	0,0014703	15,1928988	6,24282712	2,15E-10	[34;-84;-6]
						0,000141679	0,00221121	13,8630781	6,05125001	7,19E-10	[18;-96;6]
		0	3,81E-29	3663	1,81E-30	0,000155838	0,00221121	13,7626591	6,03588905	7,90E-10	[34;22;-8]
						0,006224343	0,0064947	10,3200779	5,40966459	3,16E-08	[12;14;2]
						0,037577826	0,01194703	8,91240692	5,07810972	1,91E-07	[14;10;10]
		0	4,32E-22	2412	3,08E-23	0,000759748	0,00343272	12,1821022	5,77473841	3,85E-09	[-6;32;28]
						0,002813646	0,00557716	10,9937248	5,55014633	1,43E-08	[6;20;54]
						0,011383457	0,00800356	9,8299284	5,30051011	5,77E-08	[-4;32;38]
		0	1,43E-21	2309	1,36E-22	0,002053164	0,00553207	11,2706423	5,60497637	1,04E-08	[-34;16;-10]
						0,004062381	0,0055905	10,6778908	5,48558277	2,06E-08	[-32;22;2]
						0,013812563	0,00883004	9,67673969	5,26508874	7,01E-08	[-42;18;-10]
		1,45E-06	3,45E-07	440	5,75E-08	0,063455955	0,01527139	8,53028488	4,97761379	3,22E-07	[-28;60;4]
						0,068795054	0,01582856	8,47241306	4,96194937	3,49E-07	[-46;50;0]
						0,324961758	0,04682087	7,14905739	4,56722153	2,47E-06	[-28;54;12]
		3,38E-11	1,12E-11	924	1,34E-12	0,096083048	0,01872936	8,23597813	4,89667568	4,87E-07	[-50;22;36]
						0,257975494	0,03779036	7,38954258	4,64463334	1,70E-06	[-42;-2;46]
						0,40599478	0,05765909	6,90694141	4,48640581	3,62E-06	[-34;4;58]
		6,86E-07	1,90E-07	470	2,72E-08	0,251449258	0,03718708	7,41578436	4,652915	1,64E-06	[24;52;0]
						0,562084126	0,07637271	6,51870728	4,35034433	6,80E-06	[26;48;-10]
						0,638033403	0,0888149	6,34670305	4,28734086	9,04E-06	[36;54;6]
		0,05155943	0,011005957	99	0,002096	0,65573243	0,09085623	6,30703497	4,27256159	9,66E-06	[-50;-32;-8]
						0,998893371	0,34923182	4,82889795	3,64477684	0,00013381	[-58;-30;-6]
		0,95122379	0,483524098	20	0,119618	0,999991652	0,53374951	4,40436077	3,43176359	0,00029984	[34;56;24]
		0,82980971	0,327266695	28	0,070129	0,999999318	0,62573469	4,25100422	3,35058539	0,0004032	[-2;-28;24]

Mental rotation task

Statistic: p-values adjusted for search volume

set-level		cluster-level				peak-level					
p	*c*	*p(FWE-corr)*	*p(FDR-corr)*	*kE*	*p(unc)*	*p(FWE-corr)*	*p(FDR-corr)*	*T*	*equivZ*	*p(unc)*	*x,y,z {mm}*
5,17E-14	14	4,60E-06	1,31E-06	501	2,44E-07	0,000332253	0,00770612	12,9427805	5,90520018	1,76E-09	[-48;28;24]
		0	8,81E-18	2323	4,10E-19	0,000682252	0,00770612	12,2424192	5,78543492	3,62E-09	[32;-52;64]
						0,024220244	0,02529955	9,20868969	5,15270429	1,28E-07	[38;-44;64]
						0,125309765	0,06300866	7,83571005	4,78124276	8,71E-07	[16;-62;60]
		0	3,98E-28	4609	9,26E-30	0,001795534	0,00790363	11,3508329	5,62055678	9,52E-09	[-38;-54;54]
						0,002540649	0,00790363	11,0443258	5,56028662	1,35E-08	[-30;-58;46]
						0,009112491	0,01552123	9,97294617	5,33300487	4,83E-08	[-20;-62;58]
		1,84E-08	1,40E-08	797	9,74E-10	0,002702686	0,00790363	10,9904432	5,5494868	1,43E-08	[-24;0;52]
						0,166101267	0,07566901	7,56179953	4,69842047	1,31E-06	[-22;6;58]
						0,999812064	0,61837233	4,41655922	3,43812118	0,00029288	[-20;6;72]
		4,42E-06	1,31E-06	503	2,34E-07	0,010026028	0,01552123	9,89620495	5,31563665	5,31E-08	[30;-2;58]
						0,873754693	0,25125013	5,53135729	3,96314912	3,70E-05	[30;10;60]
						0,892910488	0,25177468	5,47079897	3,93723272	4,12E-05	[24;12;50]
		0,01523698	0,003498131	160	0,000814	0,011093691	0,01552123	9,81541729	5,29718234	5,88E-08	[-30;-44;-34]
						0,956742038	0,3194611	5,20261097	3,8190987	6,70E-05	[-26;-42;-42]
						0,959487681	0,32063393	5,18639851	3,81177633	6,90E-05	[-38;-56;-34]
		0,00013774	3,49E-05	343	7,30E-06	0,093401223	0,05211752	8,12087917	4,86413421	5,75E-07	[-48;6;28]
						0,603875946	0,17229447	6,17724609	4,22353402	1,20E-05	[-40;2;32]
						0,995748403	0,43930327	4,77001524	3,61621807	0,00014947	[-50;6;18]
		1,65E-07	7,51E-08	674	8,74E-09	0,289556268	0,11396193	7,00976181	4,52108996	3,08E-06	[56;-60;-8]
						0,325713486	0,12229152	6,88835382	4,48007716	3,73E-06	[44;-60;-10]
						0,785204169	0,23217487	5,76757717	4,06169357	2,44E-05	[46;-56;2]
		7,38E-07	2,80E-07	594	3,91E-08	0,366345519	0,12342926	6,76428604	4,43736646	4,55E-06	[8;-80;-30]
						0,538990876	0,15487667	6,32375622	4,27880306	9,40E-06	[-6;-78;-38]
						0,842499374	0,24723789	5,62106848	4,00104575	3,15E-05	[26;-72;-50]
		9,54E-08	5,44E-08	704	5,06E-09	0,389628665	0,12342926	6,6978693	4,41416165	5,07E-06	[14;34;28]

				0,577126387	0,1679479	6,23703671	4,24624884	1,09E-05	[0;24;40]
				0,599157374	0,17229447	6,18774748	4,22753967	1,18E-05	[2;14;56]
0,06070773	0,01189046	115	0,003318	0,666635385	0,19362352	6,03840876	4,16991948	1,52E-05	[34;-58;-26]
				0,848182658	0,24723789	5,60542154	3,99447818	3,24E-05	[30;-66;-28]
0,4512016	0,097644529	54	0,031791	0,727967618	0,21620437	5,90133381	4,11575901	1,93E-05	[-14;-54;-46]
0,16265296	0,031110217	85	0,009405	0,844992091	0,24723789	5,61423731	3,99818064	3,19E-05	[46;34;18]
0,02610938	0,00547953	142	0,001402	0,892940209	0,25177468	5,47070074	3,93719046	4,12E-05	[36;22;-6]

Landmark task

Statistic: p-values adjusted for search volume

set-level		cluster-level				peak-level					
p	*c*	*p(FWE-corr)*	*p(FDR-corr)*	*kE*	*p(unc)*	*p(FWE-corr)*	*p(FDR-corr)*	*T*	*equivZ*	*p(unc)*	*x,y,z {mm}*
4,69E-14	24	0,00478688	0,003249541	180	0,000223	0,036316335	0,30777445	8,91107368	5,07776767	1,91E-07	[-24;-2;60]
						0,999941319	0,78450685	4,42208624	3,44099699	0,00028979	[-26;-4;44]
		0	8,21E-18	2127	1,12E-19	0,0605167	0,30777445	8,5385313	4,97983611	3,18E-07	[48;-54;-6]
						0,143698942	0,35103553	7,82676411	4,77858849	8,83E-07	[46;-66;-12]
						0,185881284	0,35103553	7,57351446	4,70202974	1,29E-06	[34;-82;34]
		2,39E-08	2,70E-08	691	1,11E-09	0,278069696	0,41399601	7,16926193	4,57383269	2,39E-06	[-38;-84;16]
						0,423712896	0,41487814	6,72028923	4,42202169	4,89E-06	[-40;-92;-4]
						0,769208965	0,44084643	5,91516352	4,12127985	1,88E-05	[-30;-92;-4]
		0,24249921	0,078368287	67	0,012882	0,33597248	0,41487814	6,97258472	4,50861199	3,26E-06	[22;4;10]
						0,995653901	0,58020298	4,87855244	3,66862247	0,00012193	[28;-2;4]
		0,02617747	0,011227493	128	0,00123	0,380582578	0,41487814	6,83892536	4,46315946	4,04E-06	[-32;-32;56]
		2,44E-13	4,13E-13	1343	1,13E-14	0,422343285	0,41487814	6,72392464	4,42329361	4,86E-06	[28;-4;54]
						0,441959097	0,41487814	6,67257881	4,40526204	5,28E-06	[26;0;72]
						0,688324238	0,44084643	6,10074043	4,19414204	1,37E-05	[56;-20;52]
		6,92E-05	5,86E-05	332	3,21E-06	0,520812746	0,44084643	6,47886896	4,33590601	7,26E-06	[10;2;34]
						0,592038542	0,44084643	6,31543016	4,27569734	9,53E-06	[14;-10;40]
						0,62570652	0,44084643	6,24014616	4,24742408	1,08E-05	[8;-2;44]
		0,10607862	0,037970682	89	0,005201	0,576433378	0,44084643	6,35063934	4,28880225	8,98E-06	[-28;-14;72]
		0,94466129	0,426099297	21	0,13425	0,666126982	0,44084643	6,15025902	4,21320833	1,26E-05	[-26;-4;4]
		0,51760428	0,176315932	46	0,033814	0,756088245	0,44084643	5,94615746	4,13360626	1,79E-05	[36;34;38]
						0,99990655	0,76825958	4,46052122	3,46091355	0,00026917	[30;32;30]
		0,33894383	0,107811991	58	0,019199	0,782734493	0,44084643	5,88266802	4,10828722	1,99E-05	[-24;10;6]
		0,10607862	0,037970682	89	0,005201	0,812606688	0,44084643	5,80839872	4,07832379	2,27E-05	[12;-28;74]
						0,956291834	0,46905986	5,31166649	3,86781041	5,49E-05	[20;-12;78]
		0,01333626	0,007178166	148	0,000623	0,892037366	0,4493786	5,58125162	3,98429835	3,38E-05	[-16;-66;54]
						0,992862584	0,56484092	4,9533534	3,70414038	0,00010605	[-8;-68;58]

				0,999521724	0,70162575	4,61222219	3,53814761	0,00020147	[-14;-58;50]
0,01472975	0,007178166	145	0,000688	0,901699	0,4493786	5,54849863	3,97043559	3,59E-05	[-42;-8;58]
				0,962066331	0,48146737	5,27681684	3,85234623	5,85E-05	[-48;-18;50]
				0,999921435	0,76825958	4,44597387	3,45339208	0,00027679	[-38;-14;52]
0,74144241	0,254452323	34	0,062742	0,935974821	0,46122174	5,41423845	3,9127792	4,56E-05	[-2;-78;-36]
0,84939369	0,320507417	28	0,08781	0,953278272	0,46905986	5,32862616	3,87530178	5,32E-05	[28;52;-2]
0,74144241	0,254452323	34	0,062742	0,977269421	0,50625571	5,16255713	3,80096989	7,21E-05	[64;-18;36]
				0,999999996	0,94392125	3,88034463	3,14440269	0,00083213	[56;-16;38]
0,62668396	0,208525615	40	0,045704	0,98005755	0,50625571	5,13588476	3,78882571	7,57E-05	[-18;-102;2]
0,13274735	0,043842071	83	0,006606	0,986679274	0,54201345	5,05899286	3,75349013	8,72E-05	[-18;10;-14]
0,84939369	0,320507417	28	0,08781	0,996487742	0,58193619	4,84851408	3,6542229	0,00012898	[-56;0;40]
0,94466129	0,426099297	21	0,13425	0,996514956	0,58193619	4,84743881	3,65370598	0,00012924	[10;-38;74]
0,86556455	0,323559847	27	0,093079	0,998962704	0,6515966	4,69574547	3,57975428	0,00017196	[60;16;32]
				0,999994035	0,85080797	4,25595522	3,35324285	0,00039935	[62;12;24]
0,95433344	0,435448834	20	0,143161	0,999646703	0,71406545	4,58180666	3,52283615	0,00021348	[44;24;28]
0,58924978	0,200855725	42	0,041272	0,99972022	0,72736884	4,55914879	3,51137379	0,0002229	[16;12;-8]
				0,999999579	0,85597981	4,09781027	3,2671203	0,00054324	[14;12;-18]

Landmark task version B session 1

Statistic: p-values adjusted for search volume

**set-level**		**cluster-level**				**peak-level**					
p	*c*	*p(FWE-corr)*	*p(FDR-corr)*	*kE*	*p(unc)*	*p(FWE-corr)*	*p(FDR-corr)*	*T*	*equivZ*	*p(unc)*	*x,y,z {mm}*
0,00177442	10	0,000586381	0,00080024	274	2,96E-05	0,219853333	0,407434768	6,28363895	4,56720054	2,47E-06	[30;22;-2]
						0,927892422	0,655405154	4,8518219	3,86568194	5,54E-05	[30;30;2]
						0,998174995	0,655405154	4,31640005	3,55906665	0,00018609	[44;20;-12]
		0,143165808	0,04216007	87	0,0078074	0,373724546	0,407434768	5,89569139	4,39236755	5,61E-06	[6;22;42]
		0,266218516	0,06033046	69	0,0156412	0,402305104	0,407434768	5,8378315	4,36538914	6,34E-06	[-20;-96;-4]
		0,070220787	0,02483317	108	0,003679	0,585631734	0,523073098	5,51222658	4,20894756	1,28E-05	[-34;18;-2]
		0,001307222	0,00089231	243	6,61E-05	0,676487833	0,536025046	5,36273527	4,13439163	1,78E-05	[50;38;26]
						0,972075065	0,655405154	4,66465235	3,76154027	8,44E-05	[42;40;36]
						0,992269034	0,655405154	4,47697496	3,65386452	0,00012916	[40;40;20]
		0,454071664	0,09175235	53	0,0305841	0,899341958	0,655405154	4,93395329	3,91038733	4,61E-05	[44;2;32]
		0,044290659	0,0206017	122	0,0022891	0,923453576	0,655405154	4,86574554	3,87330286	5,37E-05	[18;-96;-4]
						0,931435512	0,655405154	4,84031868	3,85937277	5,68E-05	[20;-90;2]
						0,984576423	0,655405154	4,57092381	3,70817979	0,00010438	[12;-94;6]
		0,315696669	0,06469444	64	0,0191687	0,974149801	0,655405154	4,65163898	3,75418052	8,70E-05	[38;54;8]
		0,202119024	0,0513425	77	0,0114094	0,993354904	0,655405154	4,45812225	3,64286377	0,00013481	[-26;-70;-48]
						0,996646281	0,655405154	4,37926531	3,59647863	0,00016128	[-36;-60;-48]
		0,920453892	0,34536334	24	0,1279123	0,99991363	0,741642225	4,06899405	3,40801337	0,00032719	[48;10;24]

Landmark task version B session 2

Statistic: p-values adjusted for search volume

set-level		cluster-level				peak-level					
p	*c*	*p(FWE-corr)*	*p(FDR-corr)*	*kE*	*p(unc)*	*p(FWE-corr)*	*p(FDR-corr)*	*T*	*equivZ*	*p(unc)*	*x,y,z {mm}*
0,00611432	9	7,97E-09	1,00E-08	849	4,18E-10	0,015226747	0,049261398	8,03072739	5,24262065	7,92E-08	[38;20;-10]
						0,049579408	0,05329877	7,25692368	4,96390491	3,45E-07	[26;12;-8]
						0,168771598	0,145312458	6,44272375	4,63597728	1,78E-06	[20;6;0]
		4,18E-05	1,87E-05	398	2,19E-06	0,033202811	0,053088242	7,52224827	5,0628124	2,07E-07	[-20;14;0]
						0,273090658	0,14696748	6,10684443	4,48880789	3,58E-06	[-26;6;0]
						0,572335496	0,190780659	5,51177359	4,20872429	1,28E-05	[-28;18;10]
		4,46E-05	1,87E-05	395	2,34E-06	0,350238658	0,165286682	5,92283154	4,40493932	5,29E-06	[42;44;30]
						0,783487825	0,275114794	5,15665007	4,02865506	2,80E-05	[44;44;22]
						0,991323943	0,478206456	4,46903086	3,64923321	0,00013151	[48;26;30]
		0,006637953	0,00209422	190	0,000349	0,459613989	0,165286682	5,70665836	4,3033219	8,41E-06	[12;-74;52]
						0,992435195	0,478206456	4,45152187	3,63900432	0,00013685	[12;-66;60]
		0,025968037	0,00661869	143	0,0013789	0,800001735	0,275114794	5,12614965	4,01270724	3,00E-05	[6;14;52]
						0,999855618	0,645877835	4,08149099	3,41579181	0,00031798	[6;24;44]
						0,999989887	0,720461502	3,91360903	3,30995048	0,00046656	[10;22;34]
		0,282370894	0,05961881	69	0,0173888	0,880235081	0,299901923	4,95912361	3,92396903	4,36E-05	[28;56;-4]
						0,888489842	0,299901923	4,93911028	3,91317452	4,55E-05	[20;46;-10]
		0,646270913	0,14523405	42	0,0544628	0,923041139	0,335993293	4,84455585	3,8616981	5,63E-05	[26;-54;42]
						0,999999391	0,818212902	3,77135396	3,21795418	0,00064554	[28;-56;52]
		0,282370894	0,05961881	69	0,0173888	0,989221537	0,478206456	4,4977746	3,66596186	0,00012321	[-4;-74;-20]
		0,531596758	0,1192408	49	0,0397469	0,998053491	0,503247165	4,2998867	3,54917497	0,00019322	[50;6;30]
						0,999987748	0,720461502	3,92444682	3,3168717	0,00045516	[54;14;34]

Landmark task version C session 1

Statistic: p-values adjusted for search volume

set-level		cluster-level				peak-level					
p	*c*	*p(FWE-corr)*	*p(FDR-corr)*	*kE*	*p(unc)*	*p(FWE-corr)*	*p(FDR-corr)*	*T*	*equivZ*	*p(unc)*	*x,y,z {mm}*
0,10998362	6	7,63E-08	1,00E-07	723	4,02E-09	0,015847118	0,033055632	8,0091877	5,2352499	8,24E-08	[36;28;-6]
						0,78290575	0,522921036	5,15460968	4,02759064	2,82E-05	[44;20;6]
						0,994328084	0,680518595	4,41309071	3,61644905	0,00014934	[52;18;2]
		0,001984966	0,0008721	236	0,0001047	0,083354933	0,089390524	6,91080666	4,82920297	6,85E-07	[8;24;38]
						0,991698122	0,680518595	4,46021509	3,64408665	0,00013417	[4;24;48]
						0,999905531	0,865277289	4,04857779	3,39527128	0,0003428	[-6;14;48]
		7,55E-05	4,97E-05	373	3,98E-06	0,344024606	0,288695591	5,93327332	4,40976213	5,17E-06	[-32;22;10]
						0,666088308	0,450626208	5,35433054	4,13014714	1,81E-05	[-38;14;14]
						0,8463946	0,54973447	5,03138924	3,96265665	3,71E-05	[-42;16;2]
		0,211443428	0,07819885	78	0,0125118	0,929694125	0,627408665	4,82045031	3,84844769	5,94E-05	[38;42;22]
						0,999374503	0,865277289	4,19245386	3,48416069	0,00024684	[42;48;26]
						0,999980699	0,929096321	3,94757843	3,33160285	0,00043174	[32;32;28]
		0,888068198	0,57670097	27	0,1153402	0,950817748	0,627408665	4,74380636	3,80597029	7,06E-05	[18;4;74]
		0,952912015	0,67060691	21	0,1609457	0,999505703	0,865277289	4,17275333	3,47211339	0,00025819	[14;-64;58]

Landmark task version C session 2

Statistic: p-values adjusted for search volume

set-level		cluster-level				peak-level					
p	*c*	*p(FWE-corr)*	*p(FDR-corr)*	*kE*	*p(unc)*	*p(FWE-corr)*	*p(FDR-corr)*	*T*	*equivZ*	*p(unc)*	*x,y,z {mm}*
0,04706651	7	0,000123566	0,00014317	351	6,51E-06	0,286821247	0,329794682	6,06822205	4,4714002	3,89E-06	[32;26;0]
						0,769502562	0,329794682	5,17882156	4,04019894	2,67E-05	[36;24;8]
		0,009782151	0,00301468	177	0,0005177	0,623109819	0,329794682	5,42498255	4,16565121	1,55E-05	[46;6;32]
						0,761724104	0,329794682	5,19262028	4,04736269	2,59E-05	[54;10;24]
						0,999888684	0,769267592	4,05999422	3,40240173	0,00033398	[54;8;16]
		0,001835762	0,00106444	239	9,68E-05	0,665955898	0,329794682	5,35462379	4,13029533	1,81E-05	[42;42;20]
						0,774856751	0,329794682	5,16923714	4,03521373	2,73E-05	[42;42;30]
						0,999997367	0,858872727	3,83874273	3,2618018	0,00055353	[44;52;14]
		0,010354012	0,00301468	175	0,0005481	0,679855262	0,329794682	5,33168364	4,11868187	1,91E-05	[12;20;34]
						0,99175722	0,695213242	4,45937395	3,64359521	0,00013443	[6;16;50]
						0,998080477	0,695213242	4,29541826	3,5464937	0,0001952	[8;28;28]
		0,241357162	0,0457191	74	0,014547	0,7575476	0,329794682	5,19997215	4,05117302	2,55E-05	[-30;20;8]
		0,033730845	0,00795098	135	0,001807	0,872521886	0,414185834	4,97414398	3,93204753	4,21E-05	[10;-74;52]
						0,99843931	0,695213242	4,27517223	3,5343204	0,00020441	[18;-70;42]
		0,071615041	0,01434905	111	0,0039134	0,953848884	0,566946798	4,73117971	3,79892128	7,27E-05	[36;-80;28]
						0,997954344	0,695213242	4,30177307	3,55030629	0,00019239	[32;-76;34]
						0,998905925	0,695213242	4,24181986	3,51417797	0,00022056	[28;-72;28]
