# Supplementary material for: Comparison of fMRI paradigms assessing visuospatial processing: Robustness and reproducibility
Source: PLoS One. 2017 Oct 23;12(10):e0186344. doi: 10.1371/journal.pone.0186344 (PMC5653292; doi:10.1371/journal.pone.0186344)
Supplement: S2 Table — Detailed overview of the non-significant correlations between task performance and the LIs. (PDF) [file pone.0186344.s002.pdf]

S2: non-significant correlations

**Lateralization indices of**	**Spearman rho correlation with acquired hit rates**	
	ρ	*p-value*
Dots in space easy frontal mask	.168	.549
Dots in space easy parietal mask	.155	.581
Dots in space hard frontal mask	-.350	.200
Dots in space hard parietal mask	.292	.291
Mental Rotation frontal mask	-.208	.458
Mental Rotation parietal mask	-.272	.326
LT version A frontal mask	-.099	.726
LT version A parietal mask	-.299	.279
LT easy 1 frontal mask	.022	.927
LT easy 1 parietal mask	.371	.118
LT hard 1 frontal mask	.069	.773
LT hard 1 parietal mask	.345	.148
LT easy 2 frontal mask	.151	.525
LT easy 2 parietal mask	.124	.635
LT hard 2 frontal mask	-.431	.058
LT hard 2 parietal mask	.321	.181
